# Regulation of cell surface protease receptor S100A10 by retinoic acid therapy in acute promyelocytic leukemia (APL)^☆^

**DOI:** 10.1038/s41419-018-0954-6

**Published:** 2018-09-11

**Authors:** Ryan W. Holloway, Margaret L. Thomas, Alejandro M. Cohen, Alamelu G. Bharadwaj, Mushfiqur Rahman, Paola Marcato, Paola A. Marignani, David M. Waisman

**Affiliations:** 1 Department of Pathology, Dalhousie University, Halifax, NS B3H 1X5 Canada; 2 Proteomic Core Facility, Faculty of Medicine, Dalhousie University, Halifax, NS B3H 1X5 Canada; 3 Department of Biochemistry and Molecular Biology, Dalhousie University, Halifax, NS B3H 1X5 Canada; 4 Department of Microbiology and Immunology, Dalhousie University, Halifax, NS B3H 1X5 Canada

## Abstract

S100A10 (p11), a member of the S100 family of small dimeric EF-hand-type Ca^2+^-binding proteins, plays a role in a variety of both intracellular and extracellular processes. Previous studies have suggested that p11 is intrinsically unstable and requires binding to annexin A2 (p36) to prevent its rapid ubiquitylation and degradation. Our laboratory has shown that p11 levels are stimulated by the expression of the oncoprotein, PML/RARα. Furthermore, treatment of the APL cell line, NB4 with all-trans retinoic acid (ATRA) causes the rapid loss of p36 and p11 protein. However, the mechanism by which ATRA regulates p11 levels has not been established. Here, we show that the proteasomal inhibitor, lactacystin reversed the ATRA-dependent loss of p11, but did not cause an accumulation of ubiquitylated forms of p11, suggesting that ATRA promotes the proteasomal degradation of p11 in an ubiquitin-independent manner. ATRA treatment of MCF-7 breast cancer cells reduced p11 but not p36 transcript and protein levels, thus indicating that ATRA can regulate p11 levels independently of PML/RARα and p36. Overexpression of p36 upregulated p11 protein but not mRNA levels, indicating that p36 affects p11 post translationally. The forced expression of ubiquitin and p11 in 293 T cells resulted in ubiquitylation of p11 that was blocked by mutagenesis of lysine 57. This study highlights the complex regulation of p11 by retinoid signaling and challenges the hypothesis that ubiquitin-mediated proteasomal degradation of p11 represents a universal mechanism of regulation of this protein.

## INTRODUCTION

S100A10 (p11) is a member of the S100 family of EF-hand-type Ca^2+^-binding proteins (reviewed in ref. ^1,2^.) that catalyzes the production of the extracellular protease plasmin, and plays a major role in fibrinolysis^3^, and macrophage migration via ECM remodeling^4,5^. Also, p11 promotes invasiveness and metastasis of numerous cancers^6–9^ via increased plasmin generation. P11 overexpression in cancers has been attributed to the presence of oncogenic RAS^7^ and the promyelocytic leukemia-retinoic acid receptor-alpha (PML/RARα) oncogene present in acute promyelocytic leukemia (APL)^9,10^. Strategies to reduce p11 in cancer cells would be critical to block plasmin-dependent metastasis.

P11 is present as a heterotetramer complex with its major binding partner, annexin A2 (p36). The intracellular interaction between p11 and p36 protects p11 protein by preventing its polyubiquitylation and subsequent degradation by the proteasome^11–14^. Studies have shown that the depletion of cellular p36 results in the rapid loss of p11 protein^11,13,15,16^ and that disrupting the interaction of p11 with p36 results in the polyubiquitylation and proteasomal degradation of p11^12,17,18^. All-trans retinoic acid (ATRA), a vitamin A metabolite^19^ and RARα ligand^20^, also reduces p11 in various cell types such as bronchial epithelial cells^15^, APL^9,10^, and dendritic cells^21^, but the mechanism is not fully understood. Since agents that block p36 protein expression have been reported to cause the rapid ubiquitylation and proteasomal degradation of p11^11,12,18^, it is unclear if the ATRA-mediated loss of p11 is direct via transcriptional regulation of the p11 gene or indirect by depleting cells of p36 protein, resulting in the ubiquitylation and proteasomal degradation of p11.

ATRA and arsenic trioxide (ATO) are the most successful treatments for APL as ATRA binding directly to the RARα moiety^22^ and ATO binds directly to the PML moiety^23^ of PML/RARα, and induce the polyubiquitylation and proteasomal degradation of PML/RARα^22–25^. Although ATRA treatment results in remission, patients still harbor a small population of APL promyelocytes containing PML/RARα transcripts^26^. Considering this, it was not surprising that subsequent studies found that APL patients cured by ATRA treatment relapsed at a median of 3.5 months after achieving remission^27,28^. Numerous studies demonstrated the combined ATRA with arsenic regimens drastically reduced relapse in adult patients with APL compared to ATRA treatments without arsenic^29–31^. We demonstrated that p11 and p36 protein levels are stimulated by the expression of the PML/RARα oncoprotein, and ATRA treatment of the APL cell line, NB4, results in the loss of p11 and p36 protein levels^9^. Interestingly, ATRA was shown to reduce p11 in cells absent of PML/RARα^15,21^, indicating that the effect of ATRA on p11 expression does not depend entirely on the loss of PML/RARα and may involve the receptor of ATRA, the RARα transcription factor.

Here we examined the mechanism(s) regulating p11 expression by ATRA as well as factors that affect retinoic acid receptor activity as the PML/RARα oncoprotein. We demonstrate that ATRA affects p11 expression at both the transcriptional and post-translational levels. We present a novel mechanism for the regulation of p11, namely ubiquitin-independent proteasomal degradation. Furthermore, we show that p11 is ubiquitylated only when ubiquitin and p11 are co-overexpressed in cells, and identify the site of ubiquitylation of p11 as lysine-57.

## RESULTS

### ATRA induces ubiquitin-independent proteasomal degradation of p11 in NB4 cells

**Fig. 1:**
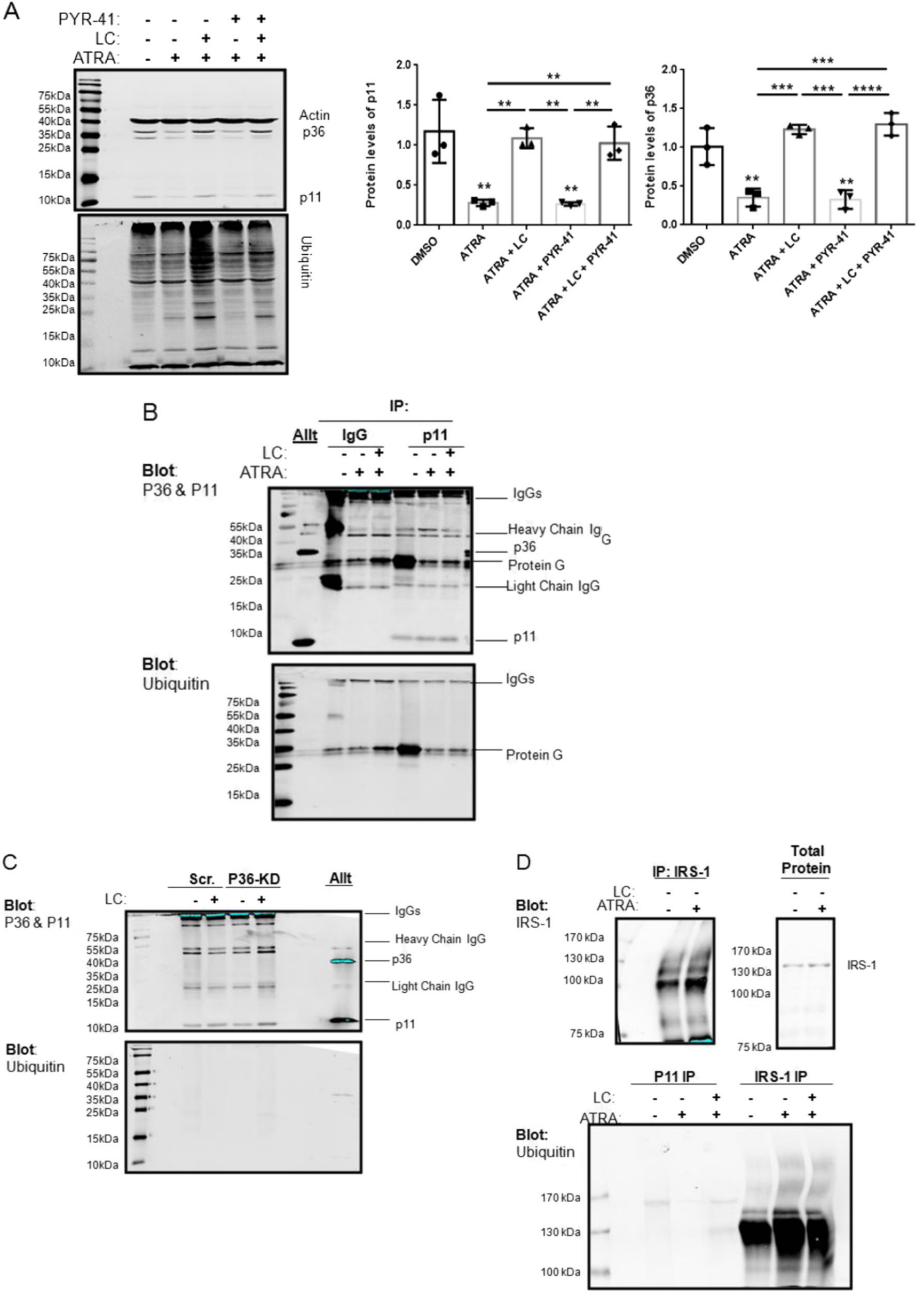
ATRA induces the ubiquitin-independent proteasomal degradation of p11 in APL cell line, NB4. **a** Immunoblot analysis of NB4 cells treated for 48 h with 1 µM ATRA alone or in combination with 2 µM lactacystin (LC) or 2 µM PYR-41. **b** Immunoprecipitation of p11 or IgG1 isotype control in NB4 cells treated for 24 h with 1 µM ATRA alone or in combination with 2 µM LC. Purified AIIt (0.25 µg) was used as a control. **c** Immunoprecipitation of p11 in PR9 cells treated for 48 h with 2 µM LC. Purified AIIt (0.25 µg) was used as a control. **d** Immunoprecipitation of p11 or IRS-1 in NB4 cells treated for 24 h with 1 µM ATRA alone, or in combination with 2 µM LC. Cell lysates were prepared and the level of the indicated proteins were examined by immunoblot analysis with β-actin used as a loading control. Data is expressed as the mean ± S.D. of three independent experiments. Statistical significance was determined using one-way ANOVA (with Tukey multiple comparisons), where **P < 0.01, ***P < 0.001, and ****P < 0.0001 are considered statistically significant

Previous studies suggested that dissociation of the p11-p36 heterotetramer complex (AIIt) by incubation of cells with plasmin or depletion of p36 by shRNA results in the ubiquitylation of p11 and its rapid degradation by the 26S proteasome^12,18^. NB4 cells are an excellent model system for studying the regulation of p11 since ATRA treatment of these cells results in the rapid loss of both p36 and p11^9,10^. NB4 cells were treated (48 h) with ATRA alone or in combination with the proteasome inhibitor lactacystin (LC), the pan-E1-ubiquitylation enzyme inhibitor PYR-41, or both. Western blot analysis of ATRA-treated NB4 cells significantly downregulated p11 and p36 expression by 3.57 ± 0.04-fold (*P* < 0.01) and 2.86 ± 0.11-fold (*P* < 0.01), respectively (Fig. 1a). PYR-41 treatments did not prevent the ATRA-dependent loss of p11; however, LC reversed the ATRA-dependent loss in p11. NB4 cells treated with ATRA and LC increased levels of ubiquitin-conjugated cellular proteins confirming that LC blocked global ubiquitin-dependent proteasomal degradation. Furthermore, the addition of PYR-41 to cells treated with ATRA and LC prevented the accumulation of ubiquitin-conjugated proteins observed with ATRA and LC treatment, confirming that PYR-41 inhibited global protein ubiquitylation. Unexpectedly, higher molecular weight species of p11 were not detected in NB4 cells treated with ATRA and LC, suggesting that ubiquitin-conjugated p11 was not present under these conditions. The ATRA-induced loss of p11 and p36 was not prevented by PYR-41, suggesting that ubiquitylation is not required for proteasomal degradation of p11. To explore this further, NB4 cells were treated with ATRA alone or in combination with LC, and p11 was immunoprecipitated and analyzed by western blotting for ubiquitin. Interestingly, ubiquitin-conjugates of p11 were not detected under these conditions, (Fig. 1b and Supplementary Fig. S1), indicating that the degradation of p11 by the proteasome did not require prior ubiquitinylated of p11.

The ubiquitylation of p11 was demonstration in p36-depleted endothelial cells, which left p11 unprotected against degradation^12^. Accordingly, p11 was immunoprecipitated from the p36-depleted cells after treatment with LC. We expected that the LC-dependent inhibition of the proteasome would result in the accumulation of ubiquitylated, unpartnered p11. The p36-depletion resulted in a loss of p11 protein, and LC treatment restored the p11 protein levels. Unexpectedly, ubiquitin-conjugated species of p11 were not detected in LC-treated, p36-depleted cells (Fig. 1c and Supplementary Fig. S2). The absence of higher molecular weight species of p11 and our inability to detect ubiquitin conjugates of p11 strongly suggested that the loss of p11 protein expression observed with p36-depleted cells was due to ubiquitin-independent proteasomal degradation, contrary to the reports that p36 protected p11 from ubiquitylation^12,17^. As a positive control, we immunoprecipitated IRS-1, an important regulatory molecule whose ubiquitylation is well established^32–34^, from NB4 cells and probed for ubiquitin. As observed in Fig. 1d, we observed a robust ubiquitylation of IRS-1, confirming that our inability to detect ubiquitinylated p11 was not due to methodological difficulties.

Ubiquitylated proteins are generally degraded by the 26S proteasome, whereas protein degradation by the 20S proteasome does not require prior ubiquitylation^35,36^. To directly investigate if p11 was a substrate for ubiquitin-independent degradation by the 20S proteasome, purified p11, p36 or AIIt proteins were incubated with a purified 20S proteasome preparation. Both p11 and p36 were degraded by the 20S proteasome (Fig. S3A–C), which was blocked by LC in vitro (Supplementary Figure S3B–C). Albumin, a negative control, was not proteolyzed under these conditions (Supplementary Fig. S3A). Together, these data support that proteasomal degradation of p11 does not require prior ubiquitylation.

### Forced co-expression of p11 and ubiquitin is required to initiate p11 ubiquitylation

**Fig. 2:**
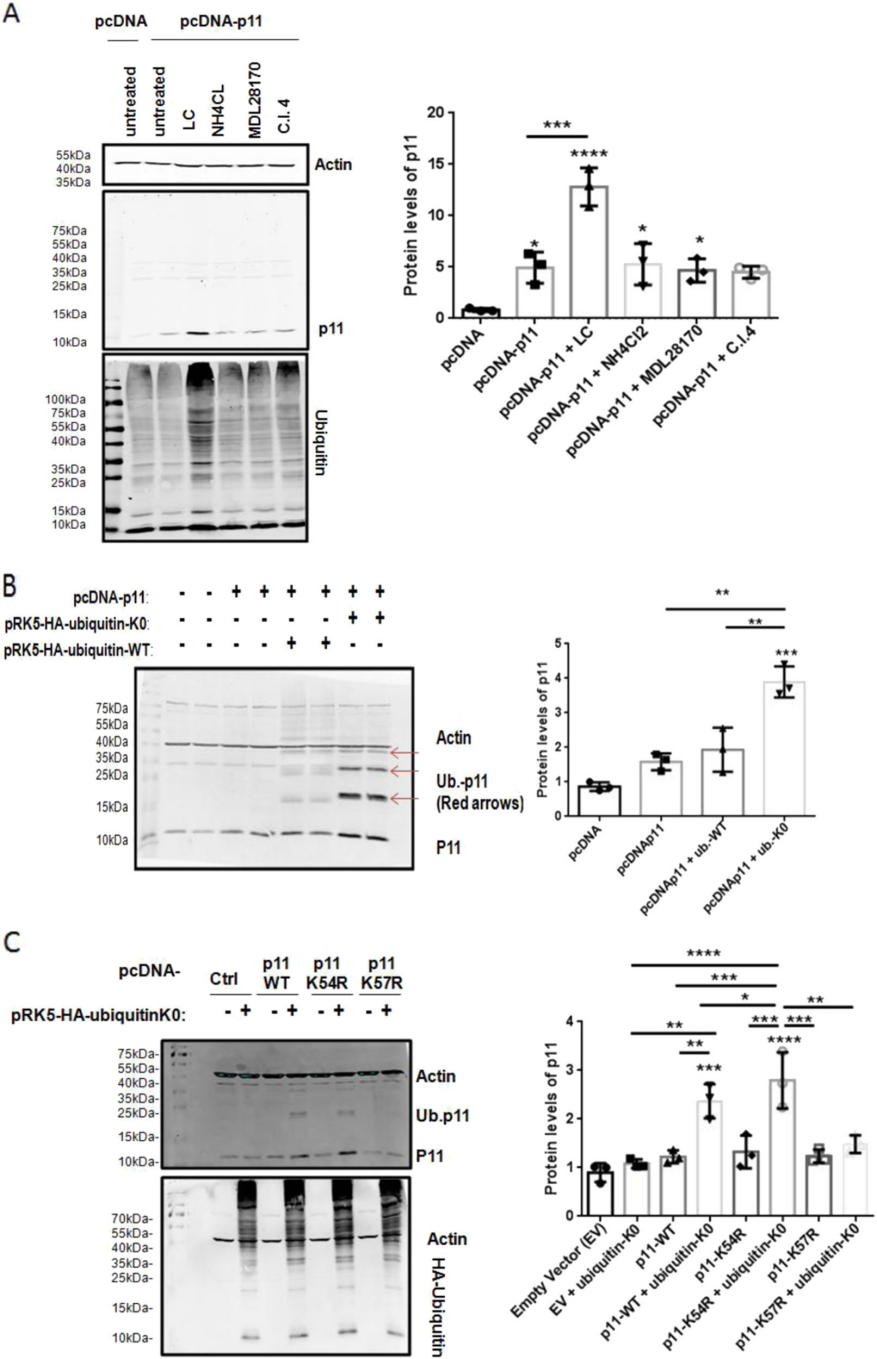
Lysine 57 is the site of ubiquitylated of p11, but may not be involved in proteasomal degradation. **a** Immunoblot analysis of HEK293T cells transiently transfected using pcDNA3.1-empty vector or pcDNA3.1-p11 vector alone or treated for 18 h using 3 µM lactacystin (LC), 1 mM NH_4_Cl, 1 µM MDL28170, or 1 µM calpain inhibitor IV (C.I.4). **b** Immunoblot analysis of HEK293T cells transiently transfected using pcDNA3.1-empty vector or pcDNA3.1-p11 vector alone or in combination with pRK5-HA-ubiquitin wild-type (ub-WT) or mutant, lysine-less ubiquitin (ub-K0). **c** Immunoblot analysis of HEK293T cells transiently expressing p11-wild type (p11WT) or p11 mutants (with Lys54 or Lys57 changed to arginine: p11K54R and p11K57R, respectively) alone or in combination with pRK5-HA-ub-K0. Cell lysates were prepared and the levels of the indicated proteins were examined by immunoblot analysis with β-actin used as a loading control. Data is expressed as the mean ± S.D. of three independent experiments. Statistical significance was determined using one-way ANOVA (with Tukey multiple comparisons), where *P < 0.05, **P < 0.01, ***P < 0.001, and ****P < 0.0001 are considered statistically significant

Ubiquitinylated p11 was initially reported in cells overexpressing both ubiquitin and p11^12,17^. HEK293T cells were transfected with a p11-expressing construct and treated with various proteolytic inhibitors. Since p36 and p11 are expressed at low levels in HEK293T cells, we reasoned that the forced expression of p11 in the presence of low intracellular levels of p36 would result in the accumulation of p11 if the appropriate proteolytic regulatory pathway were inhibited. Inhibitors of lysosomal and calpain proteolytic pathways failed to affect p11 levels. P11 accumulated only in the presence of LC, indicating that the proteasomal regulatory pathway was a key pathway for regulation of overexpressed p11 (Fig. 2a).

P11 has been shown to be ubiquitylated when co-expressed with ubiquitin in HEK293T cells^12,17^. Hence, p11 was transiently co-expressed with wild-type ubiquitin (ub-WT) or a mutant ubiquitin with all lysine residues mutated to arginine (ub-K0) to prevent the formation of polyubiquitin chains that direct proteins for proteasomal degradation^37^. Compared to the control group, HEK293T cells overexpressing p11 alone or p11 with ub-WT did not show a significant increase in p11 expression. However, co-expression of p11 and ub-K0 resulted in 4.0 ± 0.45-fold (*P* < 0.001) more p11 than control. Furthermore, higher molecular weight forms of p11 were detected only when cells were transfected with both p11 and ub-WT or ub-K0 (Fig. 2b), although the latter was more dramatic. The presence of multiple molecular weight species of p11 in cells transfected with ub-K0 suggests that p11 contains multiple ubiquitin sites. The sites of ubiquitylation on p11 were next identified by mass spectrometry using p11 immunoprecipitated from HEK293T cells co-expressing p11 and ub-K0. We observed that the ~19.5 kDa p11 band was ubiquitylated on Lys57 and the ~ 28 kDa p11 band was ubiquitylated on Lys57 and several lysines including 27 or 37 (Supplementary Fig. S4). Next, we performed solvent accessibility analysis of p11. The analysis indicated that Lys57 was 83.5% solvent exposed (Supplementary Fig. S5), making a plausible target for ubiquitylation. In order to validate the sites of ubiquitylation, ubiquitylated p11 was produced by co-transfecting HEK293T cells with vectors expressing ub-KO and p11-WT or p11 mutants. We observed that forced expression of p11-WT or p11-K54R resulted in increased p11 expression when co-expressed with ub-K0 (1.37 ± 0.21-fold, *P* < 0.001 and 2.79 ± 0.58-fold, *P* < 0.0001, respectively) and higher molecular weight forms of p11 were apparent. In contrast, co-expressing p11-K57R and ub-K0 did not significantly increase p11 levels and higher molecular weight forms were not observed (Fig. 2c). These findings strongly suggest that forced expression of p11 and ubiquitin results primarily in the ubiquitylation of Lys57 of p11.

### ATRA reduces p11 protein levels in cells expressing PML/RARa or ATRA-sensitive MCF-7 breast cancer cells

**Fig. 3:**
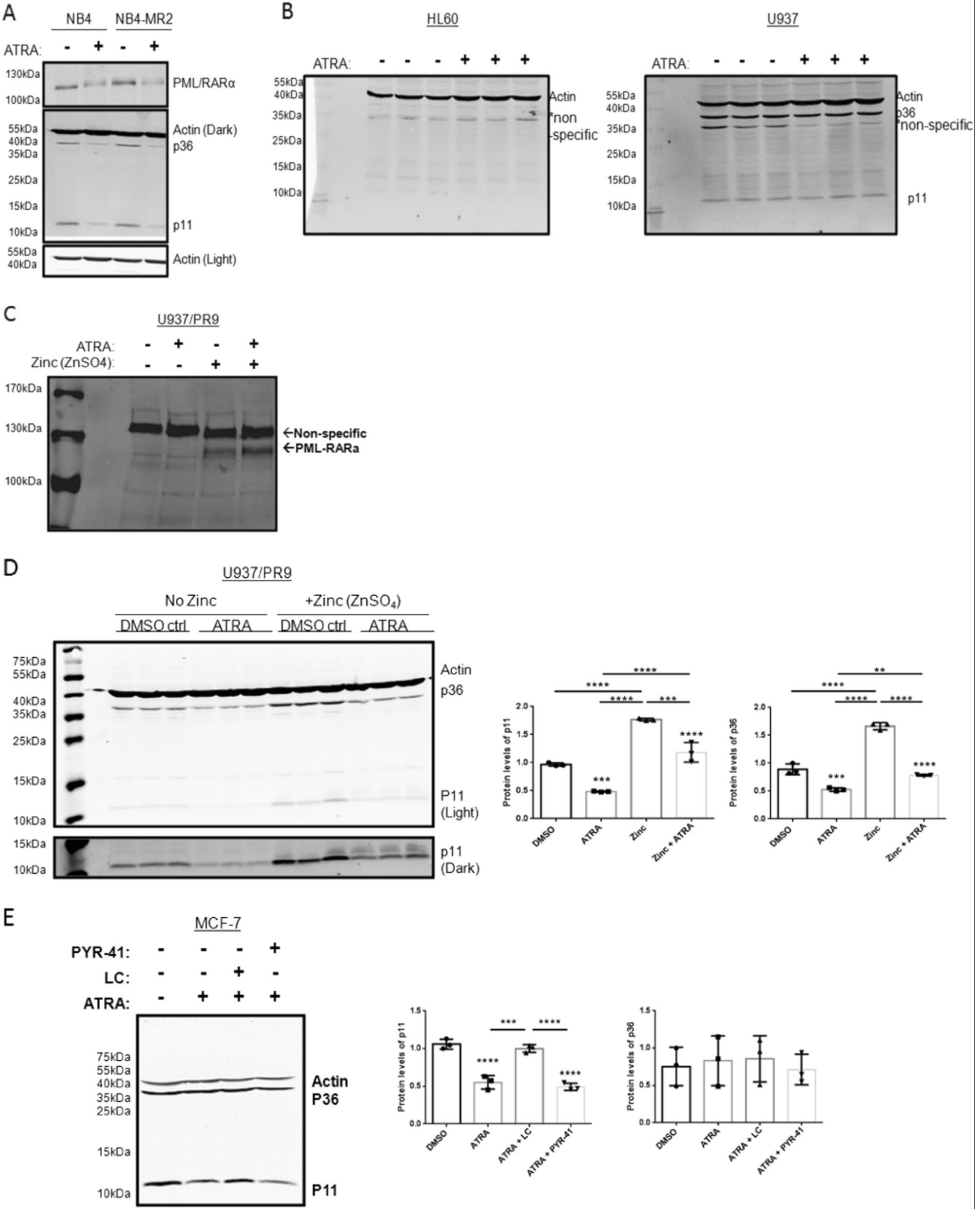
ATRA downregulates p11 and p36 expression through the loss of PML-RARα expression in APL cells. Cell lysates were prepared from (**a**) NB4 or NB4-MR2 were treated without or with 1 µM ATRA for 72 h (**b**) ATRA-treated (1 µM for 48 h) HL-60 and U937 cells, (**c**) PR9 cell treated with or without 100 µM zinc sulfate (ZnSO_4_) for 48 h, (**d**) ATRA-treated (1 µM for 48 h) PR9 ( ± 100 µM ZnSO_4_) for 48 h, and (**e**) MCF-7 cells treated for 48 h with 1 µM ATRA alone or in combination with 2 µM lactacystin (LC) or 2 µM PYR-41. Cell lysates were prepared and the levels of the indicated proteins were examined by immunoblot analysis with β-actin used as a loading control. The data are expressed as the mean ± S.D. of three independent experiments. Statistical significance was determined using one-way ANOVA (with Tukey multiple comparisons), where **P < 0.01, ***P < 0.001 and ****P < 0.0001 are considered statistically significant

ATRA treatment induces degradation of PML/RARα and allows granulocytic differentiation in NB4 cells. It is not certain whether the loss of PML/RARα or granulocytic differentiation contribute to the downregulation of p11 in ATRA-treated NB4 cells. Accordingly, the effect of ATRA on p11 and p36 expression was examined using a strain of NB4 cells resistant to ATRA-induced differentiation (NB4-MR2)^38^. ATRA-induced granulocytic differentiation was confirmed using the nitroblue tetrazolium blue (NBT) assay. As expected, 78.10 ± 9.3% of ATRA-treated NB4 cells stained positive whereas 12.45 ± 9.32% of ATRA-treated NB4-MR2 cells stained positive (Supplementary Fig. S6). Importantly, ATRA treatment (72 h) of both NB4 cell lines produced a loss of PML/RARα, p11 and p36 expression (Fig. 3a). Our previous work showed that p11 and p36 expression was absent even after 120 h of ATRA treatment of NB4 cells^9^. This suggests that p11 levels are decreased during both ATRA-induced differentiation and in cells in which ATRA does not promote differentiation. We also tested whether the other main treatment of APL, arsenic trioxide (ATO), also lead to a decrease of p11 and p36. We found that arsenic trioxide treatment (72 h) results in a significant decrease of p11 (by 2.21 ± 0.07-fold, *P* < 0.001) but not p36 (Supplementary Fig. S7). We also examined the effect of ATRA on p11 and p36 expression with other hematological cancers as AML using HL-60 cells and histiocytic lymphoma using U937 cells. Both p11 and p36 were not expressed in HL-60 cells, whereas U937 expressed both; however, ATRA treatment did not affect p11 or p36 expression in either cell line (Fig. 3b).We next tested the effect of ATRA on p11 and p36 expression using the U937-derived U937/PR9 (PR9) cell line^39^, which expresses PML/RARα on a zinc-inducible promoter. We observed that PML/RARα expression was induced, but ATRA treatment of PML/RARα-induced cells did not downregulate PML/RARα levels (Fig. 3c). This was in contrast to our observations in NB4 cells where ATRA reduced PML/RARα expression. Interestingly, under these experimental conditions, ATRA treatment reduced p11 and p36 expression in non-induced (by 2.0 ± 0.008-fold (*P* < 0.0001) and 1.69 ± 0.03-fold, (*P* < 0.001), respectively) and induced PR9 cells (by 1.49 ± 0.01-fold, *P* < 0.0001 and 2.04 ± 0.001-fold, *P* < 0.001, respectively) (Fig. 3d). This suggested that p11 and p36 expression in PR9 or U937 cells was not affected by RARα transcriptional activation. Since ATRA did not affect p11 and p36 expression in U937 cells from which the PR9 cells are derived, we reasoned that the ATRA-induced loss of p11 and p36 in the non-induced PR9 cells might be due to ‘leaky’ expression of PML/RARα from the zinc-inducible vector. We also observed that p36 levels returned to basal levels in induced PR9-p36 knockdown cells making it difficult to determine if PML/RARα increased p11 levels independent of PML/RARα-induced increases in p36 (data not shown).

To examine if ATRA could affect p11 levels in other cells, we treated MCF-7 cells with ATRA alone or in combination with LC or PYR-41. Although ATRA treatment of MCF-7 cells did not affect p36 protein levels, p11 was significantly decreased by 1.82 ± 0.09-fold (*P* < 0.01). In addition, the presence of LC, but not PYR-41, prevented the ATRA-induced loss of p11 expression in MCF-7 cells (Fig. 3e). In MCF-7 cells treated with ATRA and LC, we also did not observe higher molecular weight species of p11 suggesting the absence of ubiquitylated p11. Since ATRA inhibits proliferation of MCF-7 cells, we also confirmed that cyclin D1 was downregulated in ATRA-treated MCF-7 cells, indicating the cells responded to ATRA^40^ (Supplementary Fig. S8). The effect of ATRA on p11 expression was also assessed in other breast cancer cell lines (MDA-MB-231 and SUM159PT); however, p11 protein levels were not affected (Supplementary Fig. S9). Thus, ATRA treatment can promote p11 proteasomal degradation independently of PML/RARα and p36, although this phenomena is cell context dependent.

### ATRA regulates p11 transcription in APL and non-APL cells

**Fig. 4:**
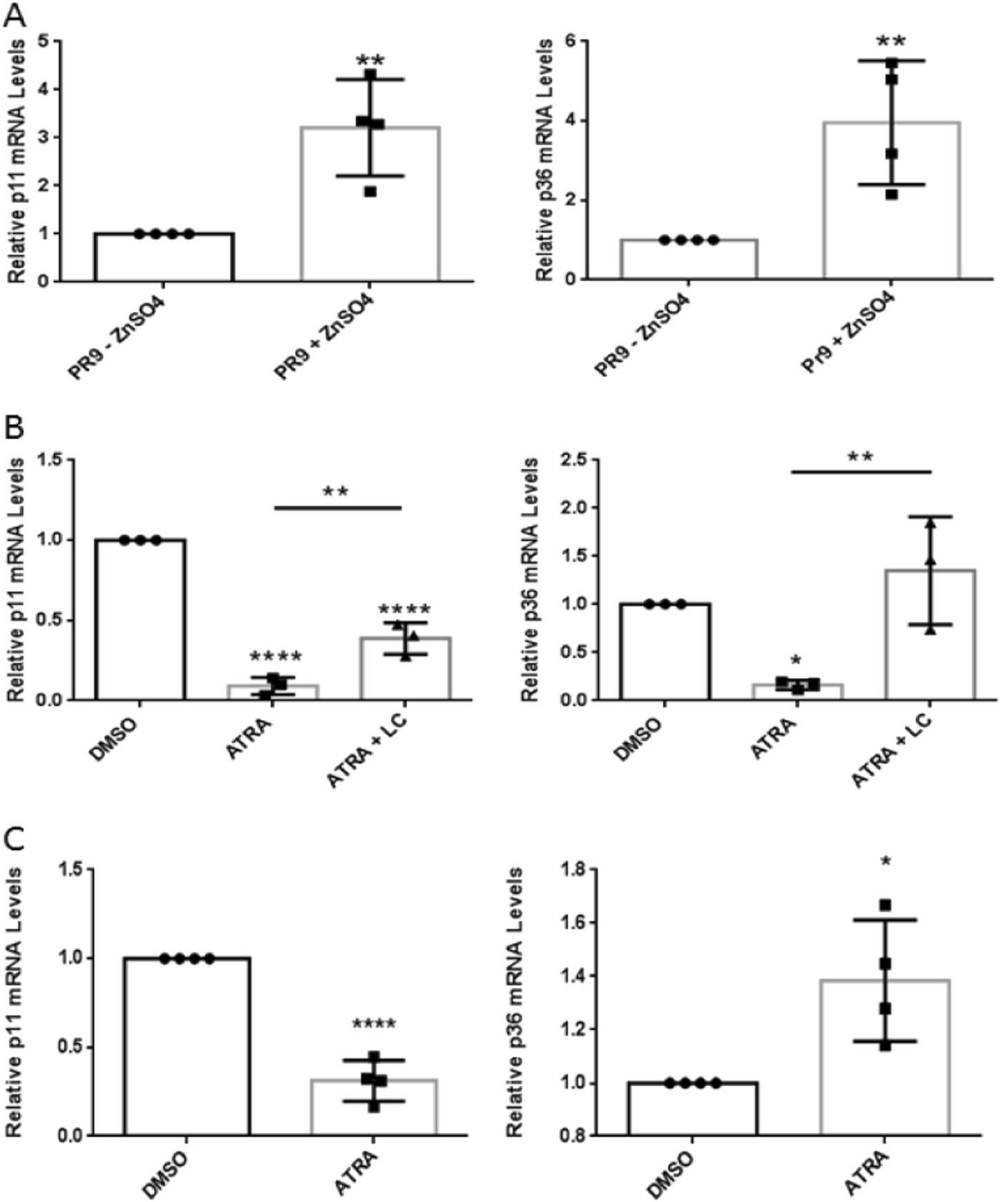
Transcript levels of p11 and p36 are regulated by PML-RARα and ATRA treatment. Total RNA extracted from (**a**) PR9 cells treated (48 h) without or with 100 µM ZnSO_4_, (**b**) NB4 cells treated (48 h) without or with 1 µM ATRA alone or in combination with 2 µM LC, and (**c**) ATRA-treated (48 h) MCF-7 cells. The relative expression of p11 and p36 mRNA levels was determined from cDNA (25 ng) by qPCR analysis and normalized to GAPDH, β-actin and HPRT1. Data is expressed as the mean ± S.D. of three (**b**) or four (**a**, **c**) independent experiments. Statistical significance was determined using (**a**, **c**) the Student *t* test for unpaired observations or (**b**) one-way ANOVA (with Tukey multiple comparisons), where *P < 0.05, **P < 0.01, and ****P < 0.0001 are considered statistically significance

ATRA treatment of NB4 cells decreases p11 protein levels, but p11 transcript levels were shown to be unaffected^9^ or reduced^41^ by the loss of PML/RARα. Induction of PML/RARα in PR9 cells resulted in the increase in p11 and p36 transcripts by 3.65 ± 0.58 (*P* < 0.01) and3.95 ± 1.56-fold (*P* < 0.01), respectively (Fig. 4a), suggesting a direct regulation of these genes by PML/RARα. Consistent with the transcriptional regulation of p11 by PML/RARα, in silico analysis of the ± 10 kb region from the p11 transcriptional start-site showed several potential binding sites for RARα and RARγ. These sites included canonical retinoic acid response element (RARE) hexameric [RGKTSA] repeats separated by 5-bp direct repeats (DR5)^42^ (Supplementary Table S1 and Supplementary Fig. S10). ATRA treatment of NB4 cells significantly reduced p11 and p36 transcript levels by 10.0 ± 0.05-fold (*P* < 0.0001) and 6.25 ± 0.05-fold (*P* < 0.0001), respectively, as compared to the control group (Fig. 4b). The ATRA-stimulated decrease in p11 transcripts was partially reversed by LC and completely reversed for p36 transcripts. LC alone did not cause a significant increase of p11 or p36 transcript levels compared to the control group (Supplementary Fig. S11). This suggested that the reversal of ATRA-mediated decreases in p11 protein by LC were due to both transcriptional and post-translational regulation. P11 transcript levels were reduced by 3.21 ± 0.11-fold (*P* < 0.0001) and p36 was increased by 1.38 ± 0.23-fold (*P* < 0.01) in ATRA-treated MCF-7 cells compared to the control group (Fig. 4c), suggesting that ATRA regulates p11 transcription possibly through interaction with the RARα receptor.

### Impact of p36 in regulating p11 protein and transcript levels

**Fig. 5:**
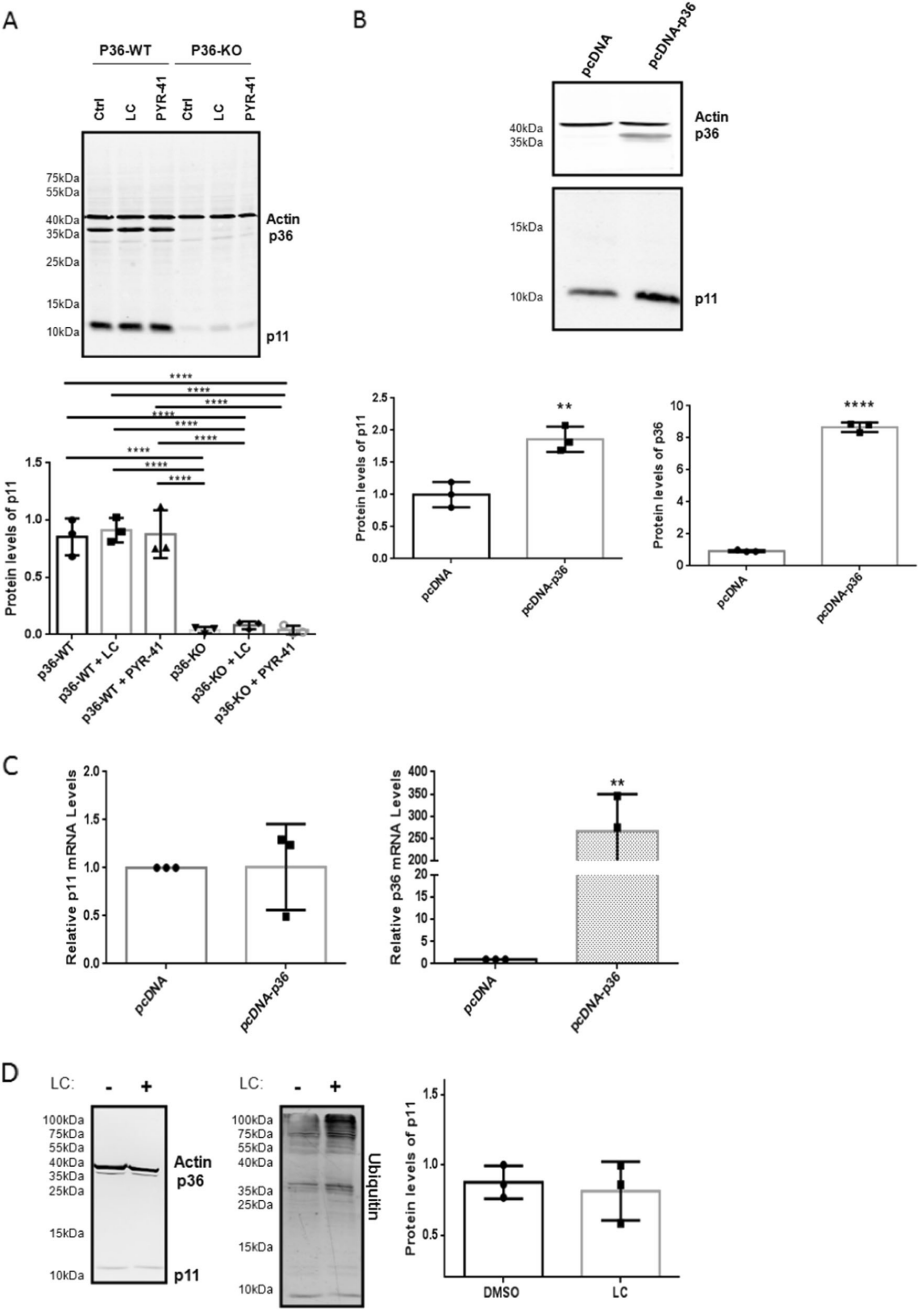
P36 upregulates p11 protein without affecting transcript levels. Immunoblot analysis of (**a**) peritoneal macrophages isolated from p36^+/+^ and p36^−/−^ mice treated for 24 h using 3 μM LC or 10 μM PYR-41 and (**b**) HEK293T cells transiently transfected using pcDNA3.1-empty vector or pcDNA3.1-p36 vector. Cell lysates were prepared and the levels of the indicated proteins were examined by immunoblot analysis with β-actin used as a loading control. Total RNA extracted from (**c**) HEK293T cells transiently transfected using pcDNA3.1-empty vector or pcDNA3.1-p36 vector were used for cDNA synthesis. The relative expression of p11 and p36 mRNA levels was determined from cDNA (25 ng) by qPCR analysis and normalized to GAPDH, β-actin and HPRT1. **d** Immunoblot analysis of HEK293T cells treated for 16 h using 2.5 µM lactacystin (LC). Cell lysates were prepared and the levels of the indicated proteins were examined by immunoblot analysis with β-actin used as a loading control. The data are expressed as the mean ± S.D. of three independent experiments. Statistical significance was determined using (**b**, **c**) the Student *t* test for unpaired observations or (**a**, **d**) one-way ANOVA (with Tukey multiple comparisons), where **P < 0.01 and ****P < 0.0001 are considered statistically significant

The simplest experimental paradigm for examining the mechanism by which p36 stabilizes p11 is to examine p11 transcript and protein levels in p36-null cells. Hence, macrophages isolated from p36-wild-type (p36^+/+^) or p36-knockout (p36^-/-^) mice were cultured in the presence or absence of LC or PYR-41 for 24 h. In Fig. 5a, p11 expression was lost due to the total depletion of p36. Surprisingly, p11 protein expression was unaffected by LC treatment, suggesting that proteasomal degradation did not contribute to the loss of p11 in p36^-/-^ cells. Next, we repeated this analysis with HEK293T cells that express low levels of p11 and p36. We reasoned that the forced expression of p36 in the presence of low intracellular levels of p11 and p36 would result in the accumulation of p11 since overexpressed p36 would be available to protect endogenous p11 from proteasomal degradation. The overexpression of p36 increased p11 protein levels by 1.86 ± 0.2-fold (*P* < 0.001) (Fig. 5b), but did not affect p11 mRNA levels (Fig. 5c). LC treatment of HEK293T cells failed to affect endogenous p11 protein levels (Fig. 5d), suggesting that endogenous protein levels of p11 were not directed to the proteasome for degradation.

## DISCUSSION

Previous studies demonstrated that either plasmin or depletion of p36 from cells resulted in the rapid ubiquitylation and proteasomal degradation of p11^11,12,17,18^. These studies also used overexpression experiments with site-directed p11 mutants to identify the carboxyl-terminal Lys91 and/or Lys93 as the sites of ubiquitylation. However, these studies failed to directly confirm the presence of ubiquitylated p11 by mass spectrometric analysis. In contrast, Wagner’s group using mass spectrometric analysis of global protein ubiquitylation reported that p11 was ubiquitylated on several internal lysines in murine tissue lysates. Our previous studies showed that PML/RARα-induction in PR9 cells upregulated protein levels of p11 and its binding and stabilizing partner, p36. With the APL cell line, NB4, we demonstrated ATRA treatment causes a dramatic reduction in p11 that was blocked by lactacystin, an inhibitor of ubiquitin-dependent and -independent proteasomal degradation. The current study had two goals: (1.) to elucidate the mechanism(s) of regulation of p11 by ATRA, and (2.) to identify the site(s) of p11 that were ubiquitylated in response to ATRA treatment, particularly with APL cells.

At pharmacological doses, ATRA converts PML/RARα from a transcriptional repressor to a transcriptional activator and induces its proteolysis^43^. Recently, it was shown that PML/RARα binds to and regulates a wider range of DNA-target sites than the canonical RARα-binding sites^44^. It has also been recently reported that PML/RARα transactivates the p36 promoter and upregulates p36 mRNA, an effect that was blocked by ATRA^45^. We observed that PML/RARα induction stimulates p11 and p36 transcription in PR9 cells, indicating that PML/RARα directly regulates p11 transcription. Furthermore, ATRA downregulated p36 and p11 levels in PR9 cells under conditions in which PML/RARα was not degraded, and ATRA did not affect p11 levels in U937 cells, the parent cell line of the PR9 cells. The simplest explanation is that in PR9 cells, PML/RARα transactivates the p11 promoter resulting in the upregulation of p11 mRNA, an effect that is blocked by ATRA. A direct effect of ATRA on p11 transcription was observed with MCF-7 cells, and ATRA was also reported to downregulate p11 in of the lung epithelial cells^15^ and dendritic cells^21^. Our data presents the possibility that ATRA inhibits p11 expression via RARα, but further experiments will be necessary to fully characterize the mechanism of the regulation of p11 by ATRA.

It is unclear if ATRA blocks the PML/RARα-induced increases in p11 in NB4 cells by blocking PML/RARα transcriptional activation at non-conical RARα-binding sites or by activating RARα-dependent inhibition of p11 transcription by activating RARα within the PML/RARα complex or by promoting the destruction of PML/RARα. This would result in increased PML/RARα-dependent stimulation of p11 and p36 transcription, and increased expression of partnered p11 protein. Our observation that p36 overexpression stimulates p11 protein levels is consistent with PML/RARα also indirectly regulating p11, post-translationally, by providing the newly translated p11 protein with its stabilizing and binding partner, p36.

The prevailing hypothesis proposes that in the absence of p36, p11 is unstable due to rapid ubiquitylation on carboxyl-terminal lysines that direct it to the proteasome for degradation^12^. The dramatic loss in p11 expression of ATRA-treated NB4 cells, and the reversal of that loss with the proteasomal inhibitor, LC suggested p11 is also regulated by ubiquitin-mediated proteasomal degradation in NB4 cells. However, this was not true and consequently, our results present a new model for the regulation for p11 protein, namely ubiquitin-independent proteasomal degradation. This model is supported by our observation that ubiquitin-conjugated p11 is not detected when p11 was immunoprecipitated from ATRA-treated NB4 cells incubated with LC, even though LC caused the accumulation of ubiquitylated proteins in ATRA-treated NB4 cells. Furthermore, when ATRA-treated NB4 cells were incubated with LC, p11 protein levels increased but ubiquitin-conjugated higher molecular weight species of p11 were not detectable. We also observed that the inhibiting protein ubiquitylation using the E1 ubiquitin-activating enzyme inhibitor, PYR-41, prevented the LC-dependent accumulation of ubiquitin-conjugated proteins, but failed to affect LC-induced increases in p11 protein. Although we cannot rule out the possibility that p11 was de-ubiquitylated during cell lysis and SDS-PAGE analysis, this would be inconsistent with our ability to detect ubiquitylated IRS1 and p11 in 293T cells overexpressing p11 and ubiquitin.

He et al. ^12^. showed that ubiquitylation was likely to involve Lys92 or Lys94 of the p11 carboxyl-terminal sequence, ^89^VHMKQKGKK^97^ by overexpressing a series of carboxyl-terminal mutants of p11. These experiments consisted of immunoprecipitation of cellular proteins with ubiquitin antibodies followed by immunoblotting for p11. However, the possibility that p11 bound to ubiquitylated proteins that were immunoprecipitated by the ubiquitin antibodies was never anticipated and importantly, the conjugation of p11 by ubiquitin was not confirmed with mass spectrometry. However, our approach utilized mass spectrometric analysis of overexpressed p11 and, consequently, we identified Lys57 as the primary target of ubiquitylation. Interestingly, Lys57 is a highly conserved lysine that is present in all 16 animal species that have been sequenced and is also surface exposed^46^. Wagner’s group mapped endogenous putative ubiquitylation sites of proteins isolated from murine tissues by mass spectrometry and identified Lys47, Lys54 and Lys57 as the primary ubiquitylated lysines in p11^47^, although these sites were not further analyzed by site-directed mutagenesis. It was also unclear in this study how much of the total p11 population was ubiquitylated and why only two of the five tissues examined yielded ubiquitinylated p11. Therefore, the physiological context by which ubiquitylation of p11 occurs is unclear. Our study shows that mutagenesis of Lys57 prevents the ubiquitylation and degradation of overexpressed p11 suggesting that this is the key site for ubiquitylation of overexpressed p11, although the contributions of other possible sites cannot be discounted. Whether ubiquitylation of p11 plays a role under physiological conditions is unclear as it was only observed after overexpression of both p11 and ubiquitin. As reviewed by Moriya^48^, the overexpression of proteins can produce cellular anomalies due to an overload on translation, and abnormalities in protein folding, localization and degradation, anyone of which could trigger anomalous ubiquitylation.

Our study presents an ATRA-regulated mechanism of p11 proteasomal degradation that may operate independently of the protection of p36-dependent protection of p11 from proteasomal degradation. Proteasomal degradation of proteins mainly occurs by ubiquitin-dependent degradation by the 26 S proteasome and ubiquitin-independent degradation by the 20 S proteasome^49^, and LC inhibits both of these mechanisms by targeting the catalytic β-subunit of the 20 S proteasome core^50^. Intrinsically disordered regions are a critical feature of proteins degraded both in a ubiquitin-dependent and ubiquitin-independent manner by the proteasome complexes, as shown for p53 and ornithine decarboxylase (Reviewed in^51,52^). Intrinsically disordered regions have been identified in p11 as well as other members of the S100 family of proteins^53^, and Lys57 is located in the helix III region that is an intrinsically disordered region. This suggests that an intrinsically disordered region around Lys57 of p11 may be critical in regulating both ubiquitin-dependent and ubiquitin-independent proteasomal degradation of p11.

## Experimental procedures

### Reagents

All-trans retinoic acid (ATRA), arsenic trioxide (ATO), zinc sulfate (ZnSO_4_), MDL28170, and ammonium chloride (NH_4_Cl) were purchased from Sigma-Aldrich (Oakville, ON, Canada). 4-nitroblue tetrazolium was purchased from Fisher Scientific (Ottawa, ON, Canada). Lactacystin was purchased from Enzo Life Sciences (East Farmingdale, NY, USA). Phorbol 12-myristate 13-acetate (PMA) were purchased from Tocris (Minneapolis, MN, USA). PYR-41 was purchased from BioVision (Milpitas, CA USA). Purified 20S proteasome was purchased from Boston Biochemical (Boston, MA, USA) or Enzo Life Sciences. Calpain inhibitor IV was purchased from MilliporeSigma (Etobicoke, ON, Canada). Purified 20S proteasome was purchased from Boston Biochemical or Enzo. Ubiquitin expression vectors (pRK5-HA-ubquitin-wild-type and –K0) were purchased from Addgene.

### Plasmids

The cDNA for full-length *p11* was amplified by PCR and ligated into the pcDNA3.1/neomycin (pcDNA-p11) vector (Invitrogen) that constitutively expresses high levels of p11 under the control of the SV40 promoter.

### Site-directed mutagenesis of p11

Site-directed mutagenesis of the pcDNA-p11was performed using the QuikChange II Site-Directed Mutagenesis Kit (Agilent Technologies) according to manufacturer’s instructions, and primers were designed using the QuikChange^®^ Primer Design Program. Mutants of p11 were produced using the following primers sets:

**Table:** 

P11 Lys54→Arg (K54R)
Fwd– 5′-CCAGGTCCTTCATTATTCTGTCCACAGCCAGAGGG-3′
Rvs– 5′-CCCTCTGGCTGTGGACAGAATAATGAAGGACCTGG-3′
P11 Lys57→Arg (K57R)
Fwd– 5′-TGGTCCAGGTCCCTCATTATTTTGTCCACAGCCAGA-3′
Rvs – 5′-TCTGGCTGTGGACAAAATAATGAGGGACCTGGACCA-3

### Cell culture

NB4 cells (DSMZ), HL-60 cells (ATCC), U937 cells (ATCC), U937/PR9 (PR9) cells, and NB4-MR2 cells (kindly provided by Dr. Wilson Miller Jr., McGill University, Montreal, QC) were maintained in RPMI-1640 medium (Invitrogen) supplemented with 10% FBS and 1% penicillin/streptomycin. MCF-7, MDA-MB-231, SUM159PT cells (kindly provided by Dr. Paola Marcato, Department of Pathology, Dalhousie University, Halifax, NS) and HEK293T cells (ATCC) were maintained in DMEM (Invitrogen) supplemented with 10% FBS and 1% penicillin/streptomycin. Non-adherent cells were maintained with the cell density kept at < 1 × 10^6^ cells/mL.

### Mice

The p36-deficient mice (p36^-/-^) and their wild-type counterparts (p36^+/+^) are on a 129SV x C57BL/6 background, and were a generous gift from Dr. K. Hajjar (Cornell University, Ithaca, NY)^54^. Experimental mice were typically 6–8 weeks of age and comprised both sexes. All animal experiments were performed in accordance with protocols approved by the University Committee on Laboratory Animals at Dalhousie University.

### Peritoneal Macrophage Isolation

Peritoneal macrophages were isolated according to Holloway et al. ^55^.

### Treatment with ATRA

NB4 and PR9 cells were seeded at a density of 0.3 × 10^6^ cells/mL and were exposed to ATRA after 24 h. MCF-7 cells were seeded to 6-well plates using 0.25–0.35 × 10^6^ cells per well and were exposed to ATRA after 24 h. Stock solutions of ATRA were diluted in DMSO (Sigma-Aldrich) and added to medium at a final concentration of 1 μM. Cells were grown in medium for the indicated times with ATRA or vehicle control added to media daily. NB4 cell density was maintained at <1 × 10^6^ cells/mL throughout the experiment.

### Zinc-Induction of PML/RARα in PR9 cells

PR9 cells (seeded at 0.3–0.5 × 10^6^ cells/mL) were treated with 100 μM ZnSO_4_ daily. Cell density was maintained at <1 × 10^6^ cells/mL throughout the experiment.

### Transient transfection

HEK293T cells were seeded in 6-well plates (2.0 × 10^5^ cells/well) and transfected the following day using the Lipofectamine2000™ transfection regent (Invitrogen) in serum-free OPTI-MEM medium (Invitrogen), according to manufacturer’s instructions.

### Immunoblot analysis and immunostaining

Cells were lysed with RIPA lysis buffer [1% Triton-X-100, 150 mM NaCl, 50 mM Tris-HCl, 1 mM EDTA, and proteinases and phosphatase inhibitor cocktails (1× final concentration; Thermo Scientific), pH 7.4]. Total protein of cell lysates (40 µg) were resolved by SDS-polyacrylamide gel electrophoresis (PAGE) using 10–20% gels (or 5% gels for PML/RARα immunoblot) and electrotransferred onto nitrocellulose membranes. The following antibodies were used for immunoblotting: p11, p36 (BD Biosciences), β-actin (Sigma), RARα (Santa Cruz, C-20), ubiquitin (Cell Signaling) and the secondary antibodies IRdye-800 goat anti-mouse antibody (LI-COR Biosciences) and IRdye-680 goat anti-rabbit antibody (Thermo Fisher). Antibody complexes were viewed on the Odyssey IR imaging system (LI-COR Biosciences). Protein expression was quantified using Image J software (NIH), and normalized to β-actin as an internal reference.

### Immunoprecipitation

For immunoprecipitation, cell were lysed in cell lysis buffer [150 mM NaCl, 50 mM Tris-HCl (pH 7.5), 1% NP-40, 1 mM phenylmethysulfonyl fluoride (PMSF), 5 mM EDTA, and complete EDTA-free protease and inhibitor cocktail (Thermo)] and 200 µg of precleared cell lysates were incubated with antibodies for mouse IgG1(R&D) or p11 (BD) for 1 h at 4 °C. Afterwards, the lysates were incubated using protein G-agarose or protein A agarose (Santa Cruz) beads for 1 h at 4 °C to collect immune complexes (antibody bound to the target protein). The beads were washed four times in cell lysis buffer, and the immune complexes were eluted from the beads by addition of 40 µL 2× SDS sample buffer and incubation at 50 °C for 10 min. The supernatants of the eluted samples were then used for western blot analysis.

### In vitro proteasomal degradation assay

The ability of the 20 S proteasome to degraded purified recombinant proteins was assessed using the 20 S proteasome assay kit (Boston Biochemical) according to manufacturer’s protocols. Briefly, ‘reaction buffer’ was diluted to 1× and the 3% SDS ‘proteasome activation’ solution was added to the buffer at a final concentration of 0.03%. Samples were prepared in this buffer without or with 1 µg of purified 20 S proteasome alone or in combination with 250 µM lactacystin (reconstituted in deionized water [dH_2_0]). Next, 1 µg of purified recombinant human proteins (p11, p36, Bovine serum Albumin (BSA) or AIIt proteins) were added to the mixture at a final volume of 20 µL and incubated for 1 h at 37 °C. The reaction was stopped by the addition of 20 µL of 2× sample loading buffer, and then boiled in water for 5 min. The protein lysate were resolved by SDS–PAGE and analyzed by immunoblot analysis or coomassie blue staining for overnight.

### Quantitative PCR (qPCR) analysis

RIBOzol RNA extraction reagent (Amresco) was used to extract RNA from cells according to manufacturer’s instruction. Briefly, cells were lysed using 1 mL of RIBOzol and transferred to an Eppendorf tube. Next, 200 μl of chloroform was added the mixture, shaken vigorously, and incubated at room temperature for 5 min. The mixture was centrifuged at 12,000 x g for 10 min at 4 °C and then the aqueous phase was collected and used to purify total RNA using the RNeasy Mini Kit (Qiagen, Valencia, CA) according to manufacturer’s protocols. The cDNA was synthesized from total RNA (1 µg) using the QuantiTect Reverse Transcription Kit (Qiagen) according to manufacturer’s protocols. Starting with 25 ng of cDNA, the reaction as carried out using the SSO Advanced Universal SYBR Green Supermix (BioRad Laboratories) and the CFX96 Real-Time PCR Detection System (Bio-Rad) to amplify the genes of interest using the following primer sets (final concentration of 0.5 µM; IDT):

Human p36: NCBI Reference Sequence: NM_001002858.2

Forward: 5′-CAAGACCAAAGGTGTGGATG-3′

Reverse: 5′-CAGTGCTGATGCAAGTTCCT-3′

Human p11: NCBI Reference Sequence: NM_002966.2

Forward: 5′-GGACCAGTGTAGAGATGGCA-3′

Reverse: 5′-TTATCAGGGAGGAGCGAACT-3′

Human Gapdh: NCBI Reference Sequence: NM_002046.5

Forward: 5′-TCAAGAAGGTGGTGAAGCAG-3′

Reverse: 5′-CGCTGTTGAAGTCAGAGGAG-3′

Human β-actin: NCBI Reference Sequence: NM_001101.3

Forward: 5′-ACGTTGCTATCCAGGCTGTG-3′

Reverse: 5′-GAGGGCATACCCCTCGTAGA-3′

Human Hprt1: NCBI Reference Sequence: NM_000194.2

Forward: 5′-TTGCTTTCCTTGGTCAGGCA-3′

Reverse: 5′-ATCCAACACTTCGTGGGGTC-3′

Fold-change values were calculated using the ΔΔCt method^56^ and normalized to β-Actin, GAPDH, and HRTP1 expression. An unpaired *t* test was used to calculate statistical significance.

### Statistical analysis

Statistical significance was determined by Student *t* test or one-way ANOVA with Tukey multiple comparisons. Results were considered as significant if two-tailed P- values were less than 0.05. All the data are expressed as mean ± S.D.

## Electronic supplementary material

supplemental methods

supplemental figures

supplementary figure legends
