# Supplementary material for: Regulation of cell surface protease receptor S100A10 by retinoic acid therapy in acute promyelocytic leukemia (APL)☆
Source: Cell Death Dis. 2018 Sep 11;9(9):920. doi: 10.1038/s41419-018-0954-6 (PMC6134137; doi:10.1038/s41419-018-0954-6)
Supplement: Supplementary file 1 — supplemental methods [file 41419_2018_954_MOESM1_ESM.pdf]

Supplemental:

Supplemental Methods:

Reagents

All-trans retinoic acid (ATRA), arsenic trioxide (ATO), zinc sulfate ($ZnSO_4$), MDL28170, and ammonium chloride (NH_4Cl) were purchased from Sigma-Aldrich (Oakville, ON, Canada). 4-nitroblue tetrazolium was purchased from Fisher Scientific (Ottawa, ON, Canada). Lactacystin was purchased from Enzo Life Sciences (East Farmingdale, NY, USA). Phorbol 12-myristate 13-acetate (PMA) were purchased from Tocris (Minneapolis, MN, USA). PYR-41 was purchased from BioVision (Milpitas, CA USA). Purified 20S proteasome was purchased from Boston Biochemical (Boston, MA, USA) or Enzo Life Sciences. Calpain inhibitor IV was purchased from MilliporeSigma (Etobicoke, ON, Canada). Purified 20S proteasome was purchased from Boston Biochemical or Enzo. Ubiquitin expression vectors (pRK5-HA-ubquitin-wild-type and –K0) were purchased from Addgene.

Treatment with ATRA or ATO

NB4 cells were seeded at a density of 0.3 x 10^6 cells/mL and were exposed to ATRA or ATO after 24 hours. Stock solutions

of ATRA were diluted in DMSO (Sigma-Aldrich) and added to medium to a final concentration of 1 μM. Stock solutions of ATO were dissolved in 1 M NaOH and added to medium to a final concentration of 0.5 μM. Cells were grown in medium for the indicated times with ATRA, ATO, or vehicle control added to media daily. NB4 cell density was kept at < 1 x 10^6 cells/mL throughout the experiment.

Plasmids

The cDNA for full-length *p11* was amplified by PCR and ligated into the pcDNA3.1/neomycin (pcDNA-p11) vector (Invitrogen) that constitutively expresses high levels of p11 under the control of the SV40 promoter.

Site-directed mutagenesis of p11

Site-directed mutagenesis of the pcDNA-p11was performed using the QuikChange II Site-Directed Mutagenesis Kit (Agilent Technologies) according to manufacturer's instructions, and primers were designed using the QuikChange® Primer Design Program. Mutants of p11 were produced using the following primers sets:

P11 Lys54$\rightarrow$Arg (K54R)

Fwd – 5’- CCA GGT CCT TCA TTA TTC TGT CCA CAG CCA GAG GG - 3’

Rvs – 5’- CCC TCT GGC TGT GGA CAG AAT AAT GAA GGA CCT GG- 3’

P11 Lys57$\rightarrow$Arg (K57R)

Fwd – 5’- TGG TCC AGG TCC CTC ATT ATT TTG TCC ACA GCC AGA- 3’

Rvs – 5’- TCT GGC TGT GGA CAA AAT AAT GAG GGA CCT GGA CCA- 3’

Immunoblot analysis and immunostaining

Cells were lysed with RIPA lysis buffer [1% Triton-X100, 150 mM NaCl, 50 mM Tris-HCl, 1 mM EDTA, and proteinases and phosphatase inhibitor cocktails (1X final concentration; Thermo Scientific), pH 7.4]. Total protein of cell lysates (40 μg) were resolved by SDS-polyacrylamide gel electrophoresis (PAGE) using 10-20% gels (or 5% gels for PML/RARα immunoblot) and electrotransferred onto nitrocellulose membranes. The following antibodies were used for immunoblotting: p11, p36 (BD Biosciences), β-actin (Sigma), RARα (Santa Cruz, C-20), and cyclin D1 (Santa Cruz), ubiquitin (Cell Signaling) and the secondary antibodies IRdye-800 goat anti-mouse antibody (LI-

COR Biosciences) and IRdye-680 goat anti-rabbit antibody (Thermo Fisher). Antibody complexes were viewed on the Odyssey IR imaging system (LI-COR Biosciences). Protein expression was quantified using Image J software.

Immunoprecipitation

For immunoprecipitation, cell were lysed in cell lysis buffer [150 mM NaCl, 50 mM Tris-HCl (pH 7.5), 1% NP-40, 1 mM phenylmethysulfonyl fluoride (PMSF), 5 mM EDTA, and complete EDTA-free protease and inhibitor cocktail (Thermo)] and 200 µg of precleared cell lysates were incubated with antibodies for mouse IgG1(R&D) or p11 (BD) for 1 h at 4°C. Afterwards, the lysates were incubated using protein G-agarose or protein A agarose (Santa Cruz) beads for 1 h at 4 °C to collect immune complexes (antibody bound to the target protein). The beads were washed four times in cell lysis buffer, and the immune complexes were eluted from the beads by addition of 40 µL 2X SDS sample buffer and incubation at 50°C for 10 min. The supernatants of the eluted samples were then used for western blot analysis.

Nitroblue Tetrazolium (NBT) Differentiation Assay

NB4 or NB4-MR2 cells were seeded in a 6-well dish (100,000 cells/5 mL) and were treated several hours later using 1µM ATRA or DMSO as a vehicle control. Cells were treated again the following day. After five days, 250,000 cells were harvested, washed in 1X DPBS and then the cell pellet was resuspended in nitroblue tetrazolium (NBT) solution [0.2% 4-nitroblue tetrazolium, 2 µg/mL phorbol 12-myristate 13-acetate (PMA) in a volume of 1.5 mL of RPMI-1640 medium] and incubated at 37 °C for 15 min. Cells were centrifuged for 5 min at 300 x g and washed with three times with cold 1X DPBS. After the final wash, the cell pellet was resuspended in 200 µL of 1X DPBS. Cells that stained positive and negative with NBT were counted using a haemocytometer and the percentage of NBT positive stained cells was represented as %NBT positive = (NBT positive cells / Total cell number) x 100.

Purification of Recombinant p11

The pAED4.91-S100A10 construct was transformed into BL21(DE3) pLysS competent *E.coli*, expressed and purified according to Ayala-Sanmartin *et al.*[1] with modifications. Bacterial cell pellets were lysed by sonication (60 s for 3 pulses) or by French press (1000 psi). Lysis was performed in 100 mM imidazole (pH 7.5), 400 mM NaCl, 10 mM $MgCl_2$, 2 mM DTT

(Dithiothreitol) with inhibitors and centrifuged at 30,000 x g for 40 min. The supernatant was precipitated with 50% $(NH_4)_2SO_4$ and centrifuged at 27,000 x g for 20 min. The resulting supernatant was applied to a Butyl-Sepharose column (GE Healthcare Life Sciences, Piscataway NJ USA)) that was equilibrated with the same buffer. S100A10 was eluted with a linear gradient from 50% to 0% $(NH_4)_2SO_4$ in the same buffer. The eluate was dialyzed against 40 mM Tris, pH 7.4, 150 mM NaCl, 0.5 mM EGTA, 0.5 mM DTT and subjected to gel permeation chromatography on a HiLoad 16/600 Superdex 75 (GE) column. S100A10 eluted as a single peak on gel permeation chromatography.

Purification of Recombinant p36

The pAED4.91-Annexin A2 construct was transformed into BL21(DE3) pLysS competent *E.coli*, and expressed and purified according to Khanna *et al.*[2] with modifications. Bacterial cell pellets were lysed by French press (1000 psi). Lysis was performed in 20 mM imidazole pH 7.5, 150 mM NaCl, 5 mM EGTA 3 mM DTT with inhibitors and centrifugation at 30,000 x g for 40 min. The NaCl concentration was reduced to 50 mM, prior to loading the supernatant onto a DEAE (MacroPrep DE, BioRad, Hercules, CA, USA) equilibrated in 20 mM Imidazole pH 7.5, 25 mM NaCl 1mM DTT. Next, 10 mM phosphate was added to the flow through

fraction and applied to a CHT™ Ceramic Hydroxyapatite (BioRad) equilibrated in 10 mM potassium phosphate pH 7.0. Annexin A2 was eluted with a linear gradient from 0.01 to 1 M KPi pH 7.0. The eluate was dialyzed against 40 mM Tris, pH 7.4, 150 mM NaCl, 0.5 mM EGTA, 0.5 mM DTT and subjected to gel permeation chromatography on a HiLoad 16/600 Superdex 75 (GE) column.

Purification of Human Recombinant Annexin A2 Heterotetramer (AIIt)

Equimolar amounts of purified (gel filtered) recombinant human S100A10 and recombinant human Annexin A2 were mixed together and incubated on ice for 30 min. The resulting complex was gel filtered on a HighLoad™ 16/600 Superdex 200 pg Size Exclusion column (GE Healthcare Life Sciences) equilibrated with 40 mM Tris-HCl pH 7.4 150 mM NaCl 0.5 mM EGTA 0.5 mM DTT. The resulting elution profile shows a major peak consisting of recombinant human annexin A2 heterotetramer (rhAIIt), followed by minor peaks of annexin A2 and S100A10 monomers. Peak identify was confirmed by SDS-PAGE. Positively identified fractions were pooled and concentrated. Protein content determined by absorbance at 280 nm, using an extinction co-efficient 1 mg/mL of 0.68.

In vitro Proteasomal Degradation Assay

The ability of the 20S proteasome to degraded purified recombinant proteins was assessed using the 20S proteasome assay kit (Boston Biochemical) according to manufacturer's protocols. Briefly, 'reaction buffer' was diluted to 1X and the 3% SDS 'proteasome activation' solution was added to the buffer at a final concentration of 0.03%. Samples were prepared in this buffer without or with 1 µg of purified 20S proteasome alone or in combination with 250 µM lactacystin (reconstituted in deionized water [dH_20]). Next, 1 µg of purified recombinant human proteins (p11, p36, or AIIt proteins) were added to the mixture at a final volume of 20 µL and incubated for 1 h at 37°C. The reaction was stopped by the addition of 20 µL of 2X sample loading buffer, and then boiled in water for 5 minutes. The protein lysate were resolved by SDS–PAGE and analyzed by immunoblot analysis or coomassie blue staining for overnight.

Quantitative PCR (qPCR) analysis

RIBOzol RNA extraction reagent (Amresco) was used to extract RNA from cells according to manufacturer's instruction. Briefly, cells were lysed using 1 mL of RIBOzol and transferred to an Eppendorf tube. Next, 200 µl of chloroform was added the

mixture, shaken vigorously, and incubated at room temperature for 5 min. The mixture was centrifuged at 12,000 x g for 10 min at 4 °C and then the aqueous phase was collected and used to purify total RNA using the RNeasy Mini Kit (Qiagen, Valencia, CA) according to manufacturer's protocols. The cDNA was synthesized from total RNA (1 µg) using the QuantiTect Reverse Transcription Kit (Qiagen) according to manufacturer's protocols. Starting with 25 ng of cDNA, the reaction as carried out using the SSO Advanced Universal SYBR Green Supermix (BioRad Laboratories) and the CFX96 Real-Time PCR Detection System (Bio-Rad) to amplify the genes of interest using the following primer sets (final concentration of 0.5 µM; IDT):

Human p36: NCBI Reference Sequence: NM_001002858.2
Forward:5'-CAAGACCAAAGGTGTGGATG-3'
Reverse: 5'-CAGTGCTGATGCAAGTTCCT-3'

Human p11: NCBI Reference Sequence: NM_002966.2
Forward:5'-GGACCAGTGTAGAGATGGCA-3'
Reverse: 5'-TTATCAGGGAGGAGCGAACT-3'

Human Gapdh: NCBI Reference Sequence: NM_002046.5
Forward:5'-TCAAGAAGGTGGTGAAGCAG-3'

Reverse: 5’-CGCTGTTGAAGTCAGAGGAG-3’

Human β-actin: NCBI Reference Sequence: NM_001101.3
Forward:5’-ACGTTGCTATCCAGGCTGTG-3’
Reverse: 5’-GAGGGCATACCCCTCGTAGA-3’

Human Hprt1: NCBI Reference Sequence: NM_000194.2
Forward:5’-TTGCTTTCCTTGGTCAGGCA-3’
Reverse: 5’-ATCCAACACTTCGTGGGGTC-3’

Fold change values were calculated using the ΔΔCt method[3] and normalized to β-Actin, GAPDH, and HRTP1 expression. An unpaired *t*-test was used to calculate statistical significance.

Surface exposed residue analysis

The online program GETAREA[4] was used to calculate the surface exposure. GETAREA calculates the surface exposure of each amino acid as a ratio of its exposed surface area in the crystal structure. Residues were scored as surface exposed, if the ratio value exceeds 50%.

Identification of a Direct Repeat Separated by 5bp (DR5) Retinoic Acid Receptor Response Element (RARE) in S100A10 Promoter Region

A single protein-coding transcript variant was identified within the ENSEMBL (GRCh38.p10) database for human S100A10 (ENSG00000197747; S100A10-202) at Chr1:151,982,915-151,994,390 [reverse strand]; with the promoter region predicted to be located at Chr1:151,992,200-151,994,201. The FASTA sequence ±10kb from the transcription start site (Chr1:151,944,390) was examined using the MEME-suite4.12.0 Motif –based sequence analysis tools FIMO (Finding Individual Motif occurrences) and Tomtom (compares one or more motifs against a database of known motifs)[5]. Using FIMO, the TSS +/- 10kb region was scanned for the classical RARE which is a repeated hexameric motif of RGKTSA separated by 5 nucleotides (DR5) [RGKTSANNNNNRGKTSA] as well as the lesser understood DR1-4 RAREs[6]. A putative DR5 [AGGTGAGGCCAGGCTCA] was identified in the promoter region and compared against a database of known transcription factor binding motifs in MEME-suite (Tomtom; searched against Jolma2013 and JASPAR CORE 2014 vertebrates) to confirm the motif's affinity for retinoic acid receptors.

Supplemental references:

1. Ayala-Sanmartin, J., Gouache, P. & Henry, J. P. N-Terminal domain of annexin 2 regulates Ca(2+)-dependent membrane aggregation by the core domain: a site directed mutagenesis study. *Biochemistry (Mosc.)* **39,** 15190–15198 (2000).
2. Khanna, N. C. *et al.* Purification and characterization of annexin proteins from bovine lung. *Biochemistry (Mosc.)* **29,** 4852–4862 (1990).
3. Livak, K. J. & Schmittgen, T. D. Analysis of relative gene expression data using real-time quantitative PCR and the 2(-Delta Delta C(T)) Method. *Methods San Diego Calif* **25,** 402–408 (2001).
4. Fraczkiewicz, R. & Braun, W. Exact and efficient analytical calculation of the accessible surface areas and their gradients for macromolecules. *J. Comput. Chem.* **19,** 319–333 (1998).
5. Bailey, T. L. *et al.* MEME SUITE: tools for motif discovery and searching. *Nucleic Acids Res.* **37,** W202-208 (2009).
6. Lalevée, S. *et al.* Genome-wide in silico identification of new conserved and functional retinoic acid receptor response elements (direct repeats separated by 5 bp). *J. Biol. Chem.* **286,** 33322–33334 (2011).

Figure S1: Ubiquitylated p11 is not detected in NB4 cells treated with ATRA alone or in combination with LC

Immunoprecipitation of p11 or IgG2a isotype control from cell lysates (200 µg) of NB4 cells treated for 24 h with 1 µM ATRA alone or in combination with 2 µM LC. Immunoprecipitated proteins were prepared and the indicated proteins were examined by western blot analysis. Data is expressed as independent experiments.

Figure S2: Ubiquitylated p11 is not detected in p36-depleted PR9 cells with or without LC treatment

Immunoprecipitation of p11 or IgG1 isotype control from cell lysates (200 µg) of PR9 cells treated for 48 h with 2 µM LC. As a control, purified AIIt (0.25 µg) was also immunoprecipitated. Immunoprecipitated proteins were prepared and the indicated proteins were examined by western blot analysis. Data is expressed as three independent experiments.

Figure S3: The 20S proteasome degrades purified p11, p36, and the p11-p36 heterotetramer, AIIt in an ubiquitin-independent manner *in vitro*

(A) Purified p11 (1 µg), p36 (1 µg), bovine AIIt heterotetramer (1 µg), and bovine serum albumin (negative control; 1 µg) were

incubated for 1 h at 37°C in the absence or presence of the 20S proteasome (1 µg) in buffer [25 mM HEPES, 0.05 mM EDTA, pH 7.6] containing 0.03% SDS for proteasomal activation. (A) Purified p11 (1 µg), p36 (1 µg), bovine AIIt heterotetramer (1 µg) protein were incubated for 1 h at 37°C in the absence or presence of the 20S proteasome (1 µg) in buffer containing 0.03% SDS for proteasomal activation with or without LC (0.5 mM). Samples were subjected to SDS-PAGE and gels were examined by (A, B) staining overnight using coomassie brilliant blue or (C) by western blot analysis. Data is expressed as three independent experiments.

Figure S4: Lys57 identified as the site of ubiquitylation of p11

HEK293T cells were transiently transfected in 100cm^2 plates using 5 µg pcDNA3.1–p11 vector in combination with 25 µg/well of pRK5-HA-ub-K0. Cell lysates were prepared and p11 was immunoprecipitated from 2000 µg of protein. Immunoprecipitated protein was resolved by SDS-PAGE and bands for higher molecular weight species of p11 (~19.5 kDa and ~28 kDa) were excised for ESI mass spectrometry analysis. Residues on the protein fragments that harbour a diglycine modification (1xGlyGly) identify the site of ubiquitylation. Diagrams highlighting the lysine residues identified that are ubiquitylated in the amino acid sequence of p11 and an immunoblot

depicting that higher molecular weight species of p11 on a western blot and their corresponding ubiquitylation sites.

Figure S5: Surface exposure of the lysine residues of p11 that were calculated using the online program, GetArea

The GetArea online program was used to calculated the surface exposure of each amino acid of p11 (residues 1-92) as a ratio of its exposed surface area in the crystal structure. Residues are considered to be solvent exposed if the ratio value exceeds 50%.

Figure S6: NBT reduction assay on NB4 or NB4-MR2 cells

NBT reduction assay on NB4 or NB4-MR2 cells after 5-day ATRA treatment. Data is expressed as the mean ± S.D. of four independent experiments. Data is expressed as four independent experiments. Statistical significance was determined using one-way ANOVA (with Tukey multiple comparisons), where ****P < 0.0001 is considered statistically significant.

Figure S7: ATRA and arsenic trioxide (ATO) downregulates p11 expression in NB4 cells

NB4 cells were treated with 1 µM ATRA, 0.5 uM ATO, or a vehicle control for 72 h. Cell lysates were prepared and expression of the indicated proteins were examined by western blot analysis with β-actin was used as a loading control. Data is expressed as

three independent experiments. Data is expressed as mean ±S.D. of three independent experiments. Statistical significance was determined using (A) one-way ANOVA (with Tukey multiple comparisons), where *P < 0.05, **P < 0.01, and ***P < 0.001 are considered statistically significant.

Figure S8: ATRA downregulates cyclin D1 expression in MCF-7 cells

MCF-7 cells were treated with 1 µM ATRA or a vehicle control for 48 h. Cell lysates were prepared and expression of the indicated proteins were examined by western blot analysis with β-actin was used as a loading control. Data is expressed as one independent experiments.

Figure S9: ATRA does not affect p11 or p36 expression in triple negative breast cancer cell lines, MDA-MB-231 and SUM159PT

(A) MDA-MB-231 and (B) SUM195PT cells were treated with 1 µM ATRA or a vehicle control for 48 h. Cell lysates were prepared and expression of the indicated proteins were examined by western blot analysis with β-actin was used as a loading control. Data is expressed as three independent experiments.

Figure S10: RAR-bind motifs in the +/-10kb region of the p11 promoter

P11 coding sequence and promoter as identified in ENSEMBL. The sequence +/-10kb of the p11 transcription start site contains several direct repeat (DR) elements with 1-5 nucleotides separating the canonical RARE hexameric [RGKTSA] repeats; identified via FIMO (Find Individual Motif Occurrences; MEME-Suite). Height of the bar representing each DR element is indicative of how well the DR element in the sequence matches the DR sequence which was anticipated (i.e. highly significant when the sequence was a perfect match to the predicted DR element). Within the promoter region of p11, a DR5 RARE was found and (B) Tomtom (MEME-suite) analysis predicts a significant association with either/both RARG & RARA. The most likely RARG motif is illustrated in the sequence logo; the top logo is the RARG_full_2 sequence from the Jolma2013 database with the lower sequence representing the sequence found in the p11 promoter region. The repeating hexameric [RGKTSA] motif is shown within the black boxes separated by 5 nucleotides; the height of each residue is indicative of their relative frequency within the sequence.

Figure S11: LC does not upregulate p11 and p36 transcript levels in NB4 cells

Total RNA extracted from NB4 cells treated (48 h) without or with 2 µM LC was used for cDNA synthesis. The relative expression of p11 and p36 mRNA levels was determined from cDNA (25 ng) by qPCR analysis and normalized to GAPDH, β-actin and HPRT1. Data is expressed as the mean ± S.D. of three independent experiments. Statistical significance was determined using the Student t-test.

Table S1: Table of RARα binding motifs; direct repeats (DR) 1-5.
