# Supplementary material for: Regulation of cell surface protease receptor S100A10 by retinoic acid therapy in acute promyelocytic leukemia (APL)☆
Source: Cell Death Dis. 2018 Sep 11;9(9):920. doi: 10.1038/s41419-018-0954-6 (PMC6134137; doi:10.1038/s41419-018-0954-6)
Supplement: Supplementary file 2 — supplemental figures [file 41419_2018_954_MOESM2_ESM.pdf]

**Figure S1**

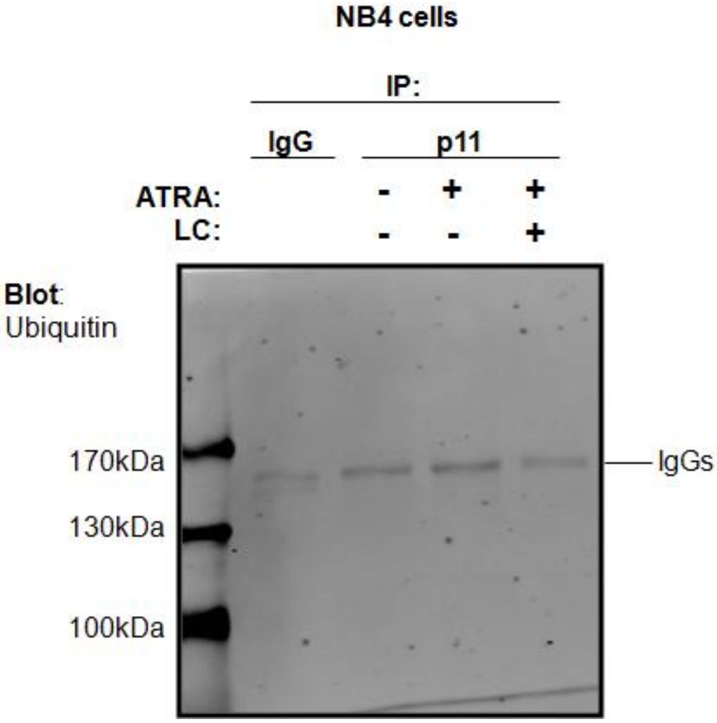
NB4 cells
IP:
IgG
p11
ATRA:
-
+
+
LC:
-
-
+
Blot:
Ubiquitin
170kDa
130kDa
100kDa
IgGs

**Figure S2**

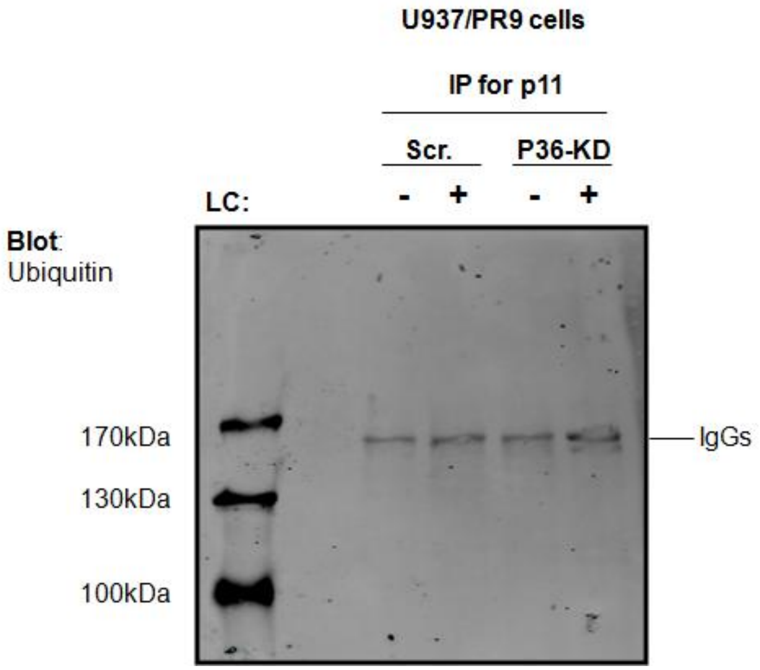
U937/PR9 cells
IP for p11
Scr.
P36-KD
LC:
-
+
-
+
Blot:
Ubiquitin
170kDa
130kDa
100kDa
IgGs

**Figure S3**

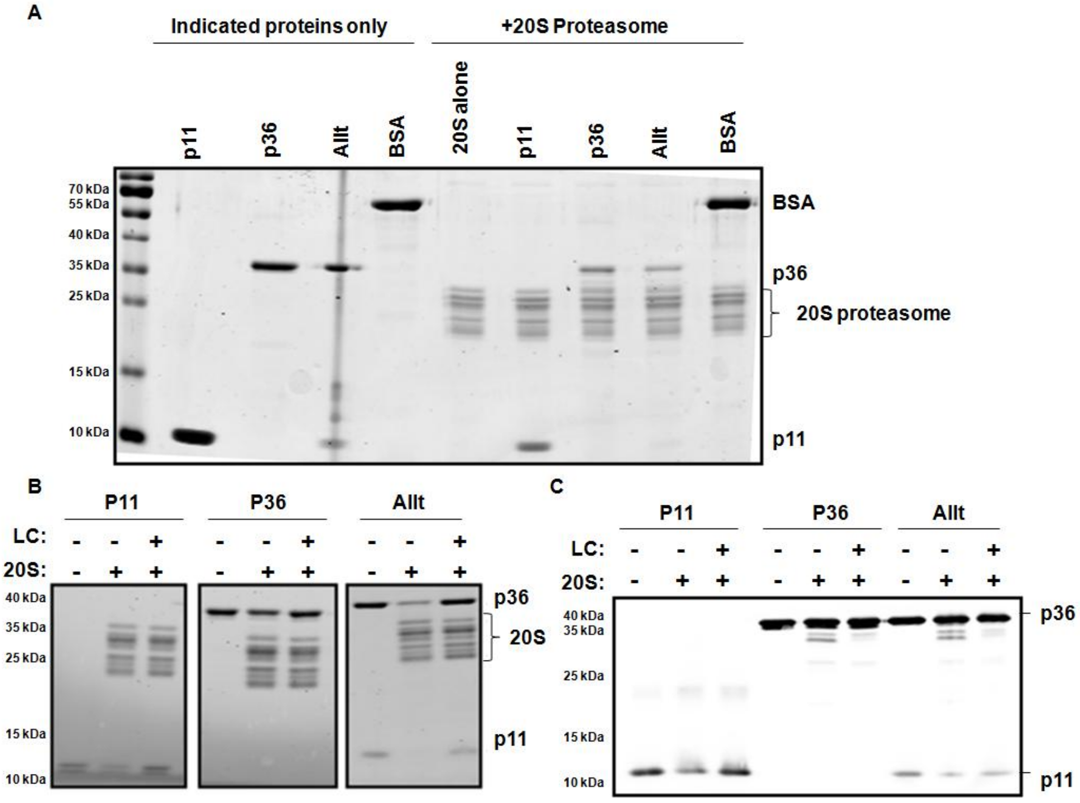
A
Indicated proteins only
+20S Proteasome
p11
p36
AIIt
BSA
20S alone
p11
p36
AIIt
BSA
70 kDa
55 kDa
40 kDa
35 kDa
25 kDa
15 kDa
10 kDa
BSA
p36
20S proteasome
p11
B
P11
P36
AIIt
LC:
20S:
40 kDa
35 kDa
25 kDa
15 kDa
10 kDa
p36
20S
p11
C
P11
P36
AIIt
LC:
20S:
40 kDa
35 kDa
25 kDa
15 kDa
10 kDa
p36
p11

**Figure S4**

## 19.5 kDa Band of p11:

| Accession | Description | |
|---|---|---|
| P60903 | Protein S100-A10 OS=Homo sapiens | |
| | Sequence | Modifications |
| | EFPGFLENQKDPLAVDK | |
| | IMKDLDQCR | 1xGlyGly [K3(100)] |
| | FAGDKGYLTK | |
| | DPLAVDK | |
| | EFPGFLENQK | |

## 28 kDa Band of p11:

| Accession | Description | |
|---|---|---|
| D3DV26 | S100 calcium binding protein A10 | |
| | Sequence | Modifications |
| | VLMEKEFPGFLENQK | 1xGlyGly [K5] |
| | EFPGFLENQKDPLAVDK | |
| | IMKDLDQCR | 1xGlyGly [K3] |
| | GYLTKEDLR | 1xGlyGly [K5] |
| | IMKDLDQCR | 1xGlyGly [K3] |
| | DPLAVDK | |
| | EFPGFLENQK | |

**Ubiquitinylation sites of p11:**

1 MPSQMEHAME
11 TMMFTFHKFA
21 GDKGYLT**K**ED
31 LRVLME**K**EFP
41 GFLENQKDPL
51 AVDKIM**K**DLD
61 QCRDGKVGFQ
71 SFFSLIAGLT
81 IACNDYFVVH
91 MKQKGKK

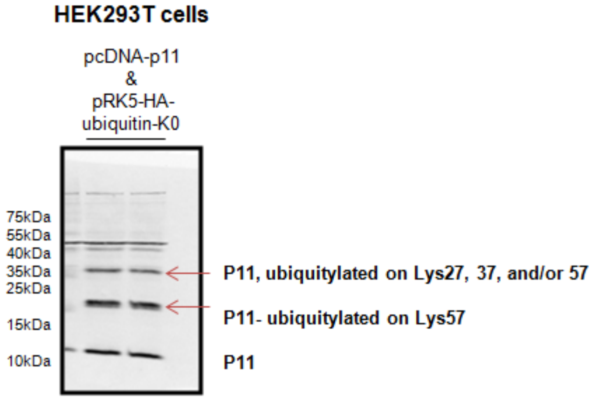

**Figure S5**

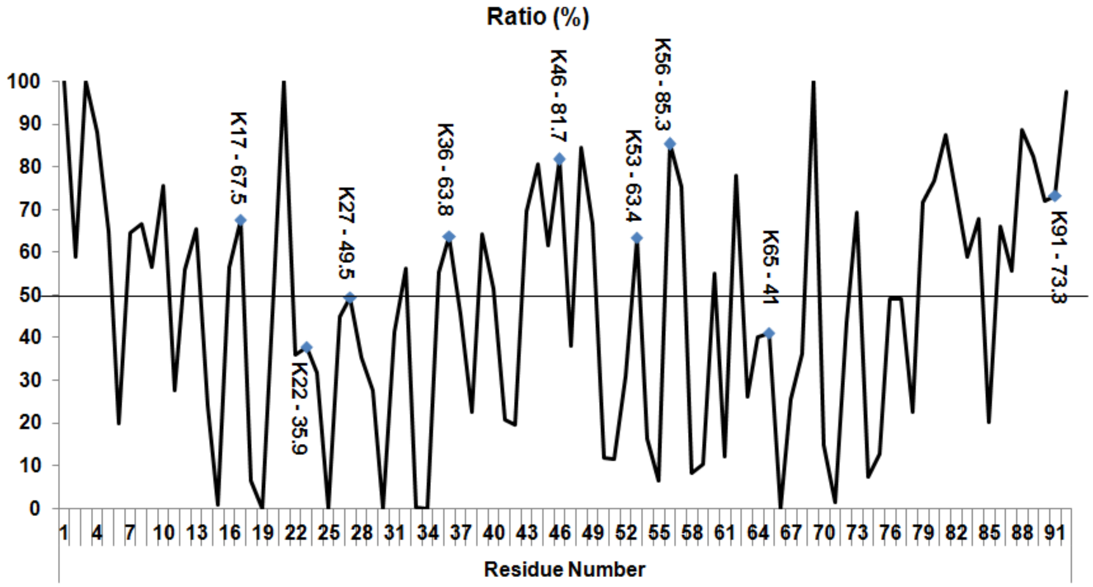

Ratio (%)
100
90
80
70
60
50
40
30
20
10
0
K17 - 67.5
K22 - 35.9
K27 - 49.5
K36 - 63.8
K46 - 81.7
K53 - 63.4
K56 - 85.3
K65 - 41
K91 - 73.3
1 4 7 10 13 16 19 22 25 28 31 34 37 40 43 46 49 52 55 58 61 64 67 70 73 76 79 82 85 88 91
Residue Number

**Figure S6**

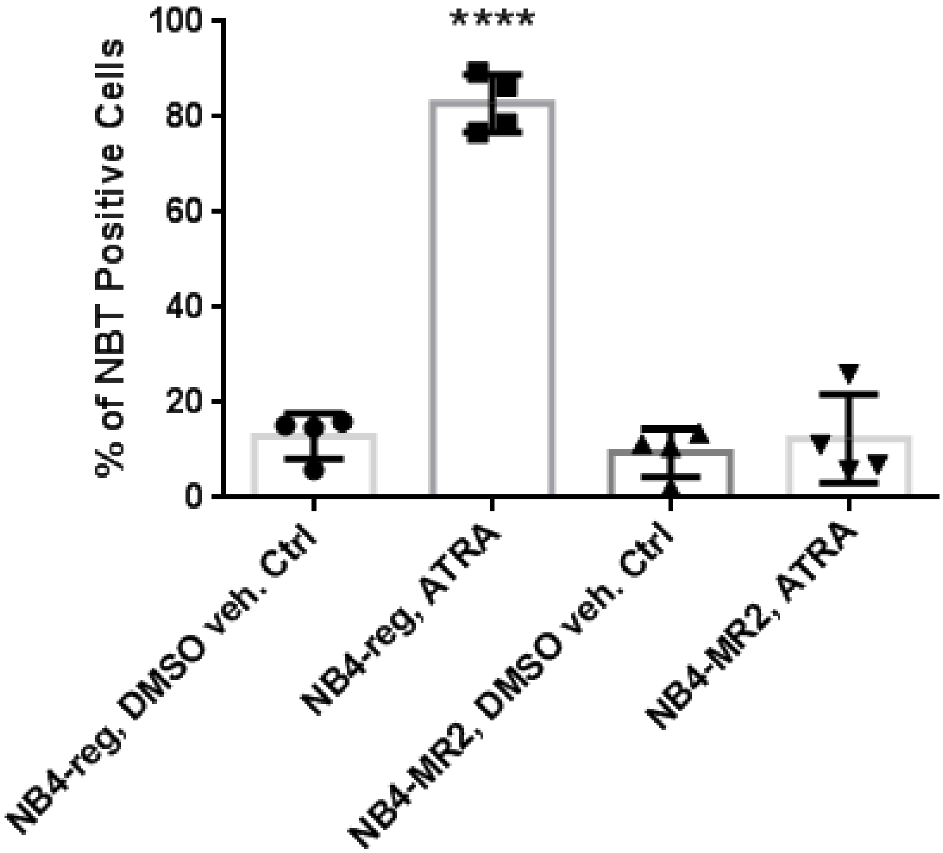
% of NBT Positive Cells
100
80
60
40
20
0
****
NB4-reg, DMSO veh. Ctrl
NB4-reg, ATRA
NB4-MR2, DMSO veh. Ctrl
NB4-MR2, ATRA

**Figure S7**

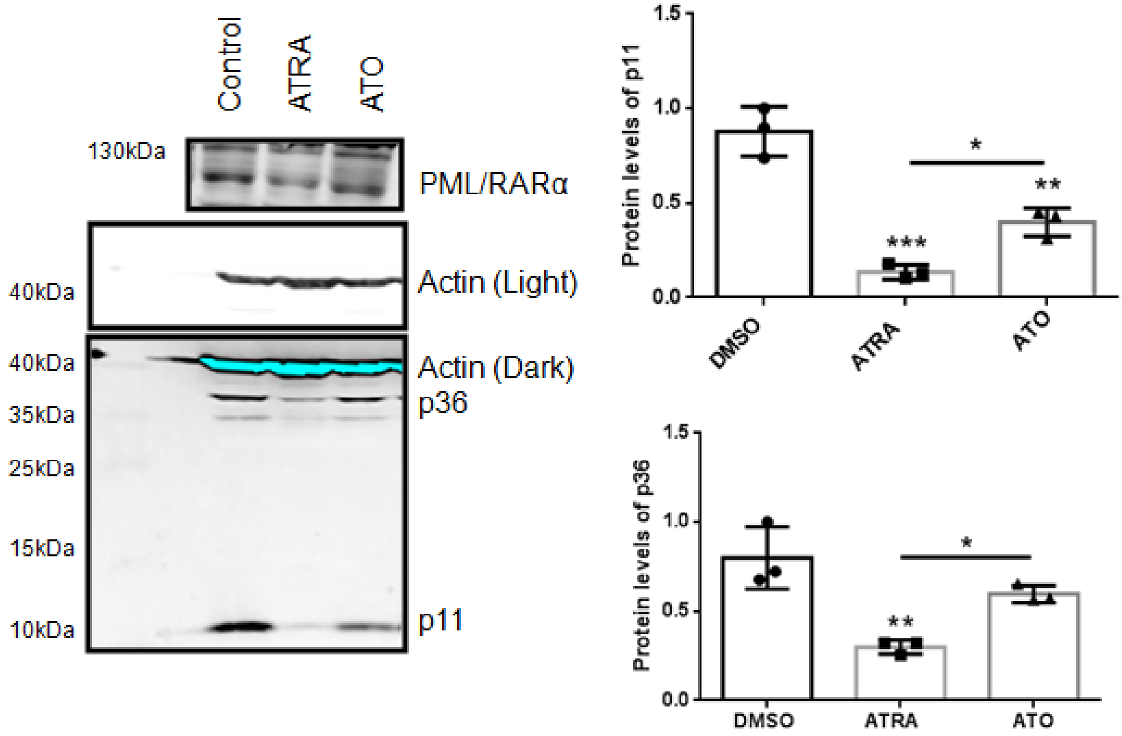
Control
ATRA
ATO
130kDa
PML/RARα
40kDa
Actin (Light)
40kDa
Actin (Dark)
35kDa
p36
25kDa
15kDa
10kDa
p11
Protein levels of p11
1.5
1.0
0.5
0.0
*
**
***
DMSO
ATRA
ATO
Protein levels of p36
1.5
1.0
0.5
0.0
*
**
DMSO
ATRA
ATO

**Figure S8**

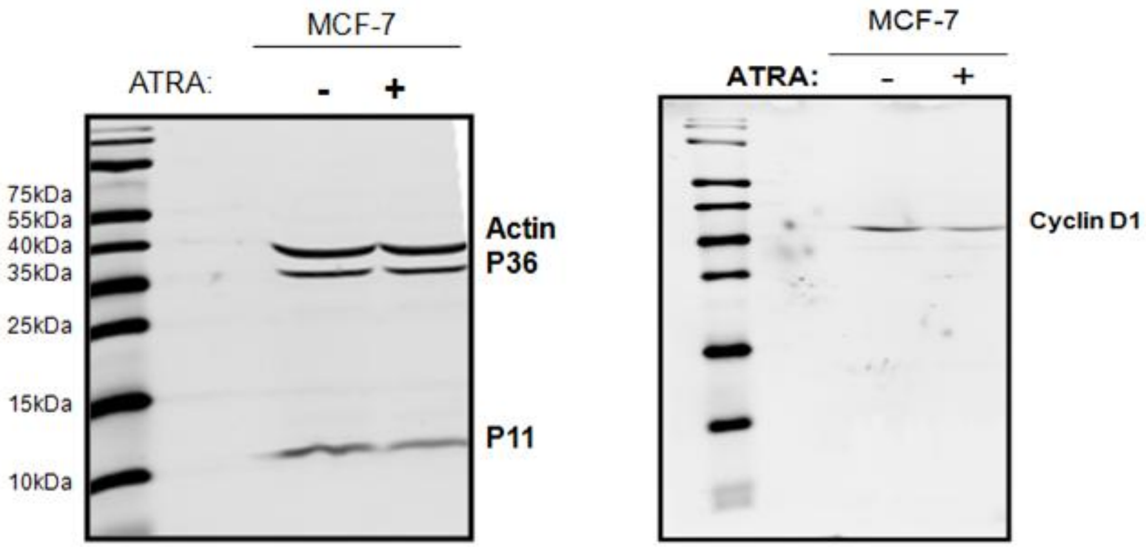
MCF-7
ATRA:
-
+
75kDa
55kDa
40kDa
35kDa
25kDa
15kDa
10kDa
Actin
P36
P11
MCF-7
ATRA:
-
+
Cyclin D1

Figure S9

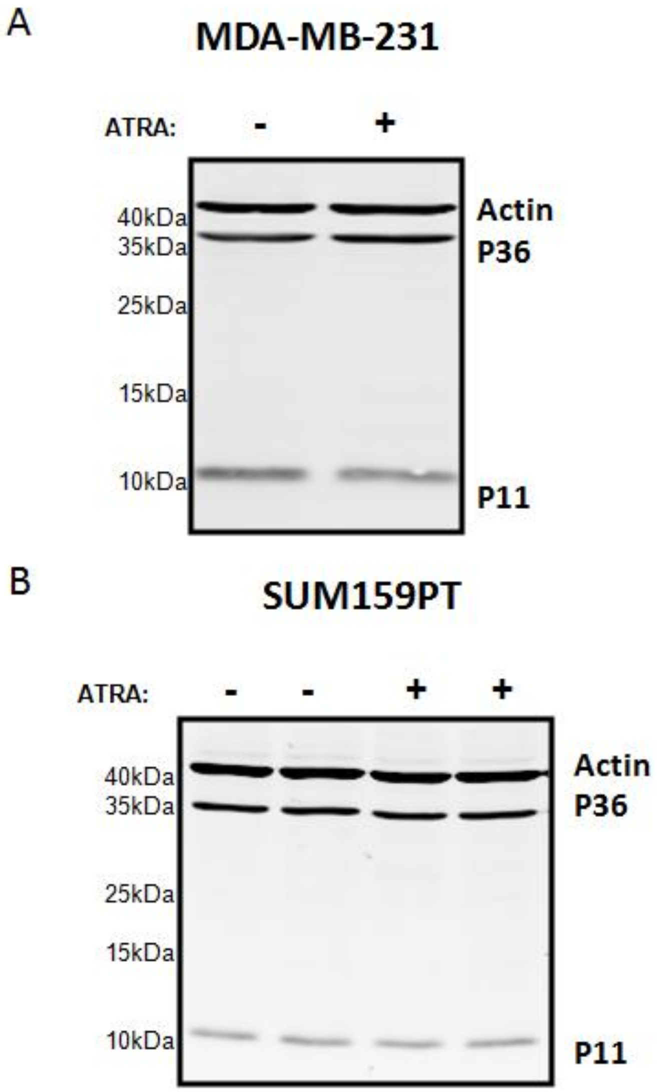
A
MDA-MB-231
ATRA:
-
+
40kDa
35kDa
25kDa
15kDa
10kDa
Actin
P36
P11
B
SUM159PT
ATRA:
-
-
+
+
40kDa
35kDa
25kDa
15kDa
10kDa
Actin
P36
P11

**Figure S10**

## A) Promoter region of S100A10

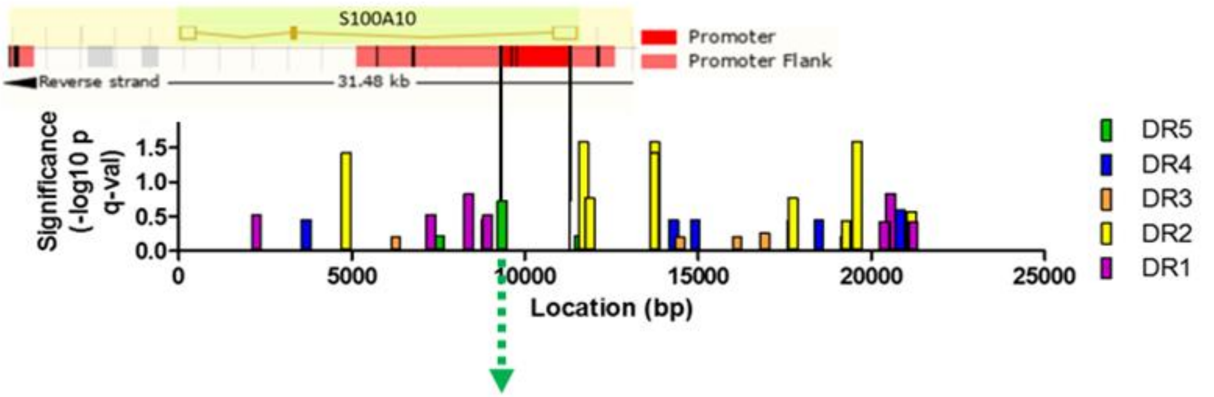


## B) DR5 element likely associates with RARG and/or RARA

| Name | RARG_full_2 | RARA_DBD_3 | RARA_full_3 | RARG_DBD_2 |
|---|---|---|---|---|
| Database | jolma2013 | jolma2013 | jolma2013 | jolma2013 |
| p-value | 0.00013 | 0.00016 | 0.00068 | 0.00335 |
| E-value | 0.181 | 0.232 | 0.981 | 4.810 |
| q-value | 0.232 | 0.232 | 0.280 | 0.711 |
| Overlap | 17 | 17 | 17 | 17 |
| Offset | 1 | 0 | 0 | 1 |
| Orientation | Normal | Normal | Normal | Normal |

**RARG_full_2**

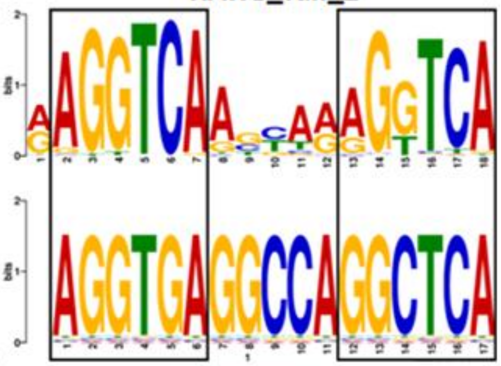

**Figure S111**

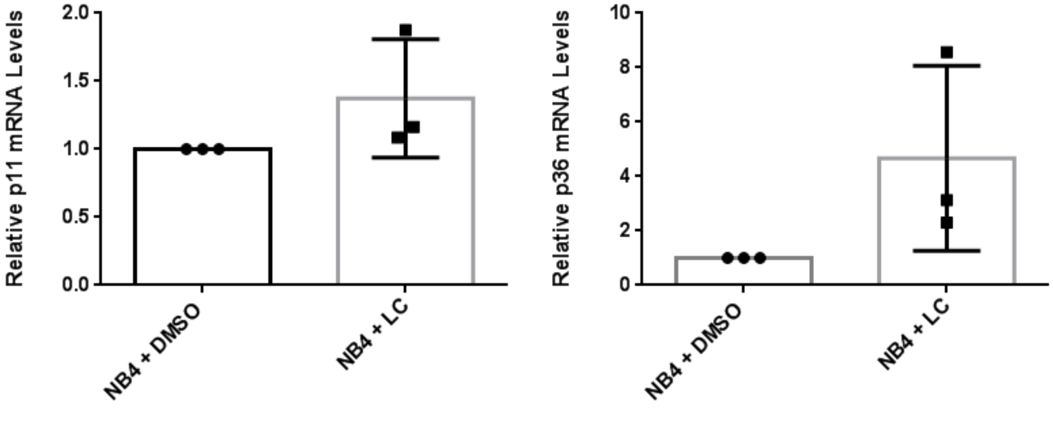
Relative p11 mRNA Levels
2.0
1.5
1.0
0.5
0.0
NB4 + DMSO
NB4 + LC
Relative p36 mRNA Levels
10
8
6
4
2
0
NB4 + DMSO
NB4 + LC

| Motif ID | Motif Sequence | Start | Stop | Strand | Score | p-value | q-value | Matched Sequence | | |
|---|---|---|---|---|---|---|---|---|---|---|
| DR5 | RGKTSAnnnnnRGKTSA | 7863 | 7879 | - | 13.2121 | 4.78E-06 | 0.19 | AGGTGA | GGCCA | GGCTCA |
| | | 19345 | 19361 | + | 11.7212 | 5.49E-05 | 0.603 | ATGTGA | GAATG | GGGTGA |
| | | 17872 | 17888 | + | 11.2303 | 7.88E-05 | 0.603 | GGTTGA | CTTTT | AAGTGA |
| | | 17783 | 17799 | + | 11.1939 | 8.11E-05 | 0.603 | AGGTGA | AAATA | GGTTGT |
| | | 6045 | 6061 | - | 10.6606 | 9.06E-05 | 0.603 | ACTTCA | ATAAA | AGGTGA |
| | | 10102 | 10118 | + | 10.6303 | 9.09E-05 | 0.603 | ACTTGA | TCAAT | AGTTCA |
| DR4 | RGKTSAnnnnRGKTSA | 10206 | 10221 | + | 13.1091 | 5.12E-06 | 0.167 | GGATCA | CCTC | AGGTCA |
| | | 18109 | 18124 | + | 12.8424 | 8.44E-06 | 0.167 | AGATCA | CCTG | AGGTCA |
| | | 19356 | 19371 | + | 12.4606 | 1.92E-05 | 0.254 | GGGTGA | AGCT | GGGAGA |
| | | 12808 | 12823 | - | 11.8727 | 4.53E-05 | 0.352 | AGGCCA | AGAT | GGGTGA |
| | | 6910 | 6925 | - | 11.6242 | 5.86E-05 | 0.352 | AGTTGG | TGAG | AGGTGA |
| | | 2214 | 2229 | - | 11.5515 | 6.90E-05 | 0.352 | GGGTGA | TTGC | ATTTGA |
| | | 16247 | 16262 | + | 11.3697 | 7.22E-05 | 0.352 | AGTAGA | TTTT | GGGTCA |
| | | 13448 | 13463 | - | 11.3515 | 7.46E-05 | 0.352 | AGTACA | GTAC | AGTTGA |
| | | 17005 | 17020 | - | 11.297 | 8.00E-05 | 0.352 | ACTTCA | CTGA | AGGTGA |
| | | 7399 | 7414 | - | 10.7697 | 9.03E-05 | 0.358 | AGTTCA | AAAA | ATGTGA |
| DR3 | RGKTSAnnnRGKTSA | 15447 | 15461 | - | 12.6545 | 1.38E-05 | 0.548 | GGTTCA | TAG | TGGTGA |
| | | 4793 | 4807 | + | 12.0424 | 3.36E-05 | 0.63 | AGGAGA | GCA | AGGTGA |
| | | 12998 | 13012 | - | 11.703 | 5.75E-05 | 0.63 | AGTTGC | TCT | GGGTGA |
| | | 14654 | 14668 | + | 11.6667 | 6.33E-05 | 0.63 | AGTTCA | CAC | AGGTCT |
| DR2 | RGKTSAnnRGKTSA | 12270 | 12283 | + | 17.0788 | 1.62E-06 | 0.0257 | AGGTCA | AC | AGGTCA |
| | | 10216 | 10229 | + | 17.0667 | 1.94E-06 | 0.0257 | AGGTCA | GG | AGTTCA |
| | | 18119 | 18132 | + | 17.0667 | 1.94E-06 | 0.0257 | AGGTCA | GG | AGTTCA |
| | | 3368 | 3381 | - | 13.1091 | 4.70E-06 | 0.0374 | GGATCA | CG | AGGTCA |
| | | 12262 | 12275 | + | 13.1091 | 4.70E-06 | 0.0374 | GGATCA | CG | AGGTCA |
| | | 10395 | 10408 | + | 12.1697 | 2.86E-05 | 0.169 | GGGTGA | CA | GAGTGA |
| | | 16257 | 16270 | + | 12.1576 | 2.98E-05 | 0.169 | GGGTCA | TT | GGGGCA |
| | | 19689 | 19702 | - | 11.8121 | 5.35E-05 | 0.266 | AGGTCA | CC | ACGTCA |
| | | 17794 | 17807 | + | 11.2788 | 8.33E-05 | 0.368 | GGTTGT | TA | AGTTGA |
| DR1 | RGKTSAnRGKTSA | 19074 | 19086 | + | 13.2606 | 3.71E-06 | 0.146 | GGGTGA | G | GGATGA |
| | | 6910 | 6922 | - | 12.9152 | 7.39E-06 | 0.146 | TGGTGA | G | AGGTGA |
| | | 779 | 791 | - | 12.303 | 2.46E-05 | 0.296 | GGGTGA | G | AGGAGA |
| | | 5812 | 5824 | - | 12.103 | 3.12E-05 | 0.296 | CGTTCA | T | AGTTGA |
| | | 7449 | 7461 | - | 12.0061 | 3.74E-05 | 0.296 | AGGTCA | G | GGTGGA |
| | | 18925 | 18937 | + | 11.6667 | 6.50E-05 | 0.375 | GGGTCA | G | ATTTGA |
| | | 19735 | 19747 | + | 11.6545 | 6.64E-05 | 0.375 | AGTTGT | C | GGTTCA |
| | | 18883 | 18895 | + | 11.4182 | 7.64E-05 | 0.378 | ACTTCA | T | GGGTGA |
