# Supplementary material for: Regulation of cell surface protease receptor S100A10 by retinoic acid therapy in acute promyelocytic leukemia (APL)☆
Source: Cell Death Dis. 2018 Sep 11;9(9):920. doi: 10.1038/s41419-018-0954-6 (PMC6134137; doi:10.1038/s41419-018-0954-6)
Supplement: Supplementary file 3 — supplementary figure legends [file 41419_2018_954_MOESM3_ESM.docx]

**Figure S1: Ubiquitylated p11 is not detected in NB4 cells treated with ATRA alone or in combination with LC**

Immunoprecipitation of p11 or IgG2a isotype control from cell lysates (200 µg) of NB4 cells treated for 24 h with 1 µM ATRA alone or in combination with 2 µM LC. Immunoprecipitated proteins were prepared and the indicated proteins were examined by western blot analysis. Data is expressed as independent experiments.

**Figure S2: Ubiquitylated p11 is not detected in p36-depleted PR9 cells with or without LC treatment**

Immunoprecipitation of p11 or IgG1 isotype control from cell lysates (200 µg) of PR9 cells treated for 48 h with 2 µM LC. As a control, purified AIIt (0.25 µg) was also immunoprecipitated. Immunoprecipitated proteins were prepared and the indicated proteins were examined by western blot analysis. Data is expressed as three independent experiments.

**Figure S3: The 20S proteasome degrades purified p11, p36, and the p11-p36 heterotetramer, AIIt in an ubiquitin-independent manner *in vitro***

(A) Purified p11 (1 µg), p36 (1 µg), bovine AIIt heterotetramer (1 µg), and bovine serum albumin (negative control; 1 µg ) were incubated for 1 h at 37°C in the absence or presence of the 20S proteasome (1 µg) in buffer [25 mM HEPES, 0.05 mM EDTA, pH 7.6] containing 0.03% SDS for proteasomal activation. (A) Purified p11 (1 µg), p36 (1 µg), bovine AIIt heterotetramer (1 µg) protein were incubated for 1 h at 37°C in the absence or presence of the 20S proteasome (1 µg) in buffer containing 0.03% SDS for proteasomal activation with or without LC (0.5 mM). Samples were subjected to SDS-PAGE and gels were examined by (A, B) staining overnight using coomassie brilliant blue or (C) by western blot analysis. Data is expressed as three independent experiments.

**Figure S4: Lys57 identified as the site of ubiquitylation of p11**

HEK293T cells were transiently transfected in 100cm^2^ plates using 5 μg pcDNA3.1–p11 vector in combination with 25 μg/well of pRK5-HA-ub-K0. Cell lysates were prepared and p11 was immunoprecipitated from 2000 µg of protein. Immunoprecipitated protein was resolved by SDS-PAGE and bands for higher molecular weight species of p11 (~19.5 kDa and ~28 kDa) were excised for ESI mass spectrometry analysis. Residues on the protein fragments that harbour a diglycine modification (1xGlyGly) identify the site of ubiquitylation. Diagrams highlighting the lysine residues identified that are ubiquitylated in the amino acid sequence of p11 and an immunoblot depicting that higher molecular weight species of p11 on a western blot and their corresponding ubiquitylation sites.

**Figure S5: Surface exposure of the lysine residues of p11 that were calculated using the online program, GetArea**

The GetArea online program was used to calculated the surface exposure of each amino acid of p11 (residues 1-92) as a ratio of its exposed surface area in the crystal structure. Residues are considered to be solvent exposed if the ratio value exceeds 50%.

**Figure S6: NBT reduction assay on NB4 or NB4-MR2 cells**

NBT reduction assay on NB4 or NB4-MR2 cells after 5-day ATRA treatment. Data is expressed as the mean ± S.D. of four independent experiments. Data is expressed as four independent experiments. Statistical significance was determined using one-way ANOVA (with Tukey multiple comparisons), where ****P < 0.0001 is considered statistically significant.

**Figure S7: ATRA and arsenic trioxide (ATO) downregulates p11 expression in NB4 cells**

NB4 cells were treated with 1 µM ATRA, 0.5 uM ATO, or a vehicle control for 72 h. Cell lysates were prepared and expression of the indicated proteins were examined by western blot analysis with β-actin was used as a loading control. Data is expressed as three independent experiments. Data is expressed as mean ±S.D. of three independent experiments. Statistical significance was determined using (A) one-way ANOVA (with Tukey multiple comparisons), where *P < 0.05, **P < 0.01, and ***P < 0.001 are considered statistically significant.

**Figure S8: ATRA downregulates cyclin D1 expression in MCF-7 cells**

MCF-7 cells were treated with 1 µM ATRA or a vehicle control for 48 h. Cell lysates were prepared and expression of the indicated proteins were examined by western blot analysis with β-actin was used as a loading control. Data is expressed as one independent experiments.

**Figure S9: ATRA does not affect p11 or p36 expression in triple negative breast cancer cell lines, MDA-MB-231 and SUM159PT**

(A) MDA-MB-231 and (B) SUM195PT cells were treated with 1 µM ATRA or a vehicle control for 48 h. Cell lysates were prepared and expression of the indicated proteins were examined by western blot analysis with β-actin was used as a loading control. Data is expressed as three independent experiments.

**Figure S10: RAR-bind motifs in the +/-10kb region of the p11 promoter**

P11 coding sequence and promoter as identified in ENSEMBL. The sequence +/-10kb of the p11 transcription start site contains several direct repeat (DR) elements with 1-5 nucleotides separating the canonical RARE hexameric [RGKTSA] repeats; identified via FIMO (Find Individual Motif Occurrences; MEME-Suite). Height of the bar representing each DR element is indicative of how well the DR element in the sequence matches the DR sequence which was anticipated (i.e. highly significant when the sequence was a perfect match to the predicted DR element). Within the promoter region of p11, a DR5 RARE was found and (B) Tomtom (MEME-suite) analysis predicts a significant association with either/both RARG & RARA. The most likely RARG motif is illustrated in the sequence logo; the top logo is the RARG_full_2 sequence from the Jolma2013 database with the lower sequence representing the sequence found in the p11 promoter region. The repeating hexameric [RGKTSA] motif is shown within the black boxes

separated by 5 nucleotides; the height of each residue is indicative of their relative frequency within the sequence.

**Figure S11: LC does not upregulate p11 and p36 transcript levels in NB4 cells**

Total RNA extracted from NB4 cells treated (48 h) without or with 2 µM LC was used for cDNA synthesis. The relative expression of p11 and p36 mRNA levels was determined from cDNA (25 ng) by qPCR analysis and normalized to GAPDH, β-actin and HPRT1. Data is expressed as the mean ± S.D. of three independent experiments. Statistical significance was determined using the Student t-test.

**Table S1: Table of RARα binding motifs; direct repeats (DR) 1-5.**
